# Downstream Effectors of ILK in Cisplatin-Resistant Ovarian Cancer

**DOI:** 10.3390/cancers12040880

**Published:** 2020-04-04

**Authors:** Jeyshka M. Reyes-González, Blanca I. Quiñones-Díaz, Yasmarie Santana, Perla M. Báez-Vega, Daniel Soto, Fatima Valiyeva, María J. Marcos-Martínez, Ricardo J. Fernández-de Thomas, Pablo E. Vivas-Mejía

**Affiliations:** 1 Department of Biochemistry, Medical Sciences Campus, University of Puerto Rico, San Juan, PR 00936, USA; 2 Center for Collaborative Research in Health Disparities (CCRHD), Medical Sciences Campus, University of Puerto Rico, San Juan, PR 00936, USA; 3 Comprehensive Cancer Center, University of Puerto Rico, San Juan, PR 00936, USA; 4 Department of Biology, Rio Piedras Campus, University of Puerto Rico, San Juan, PR 00931, USA; 5 Department of Pathology and Laboratory Medicine, Medical Sciences Campus, University of Puerto Rico, San Juan, PR 00936, USA; 6 Anatomic Pathology Laboratory, Puerto Rico Medical Services Administration, San Juan, PR 00935, USA; 7 School of Medicine, Medical Sciences Campus, University of Puerto Rico, San Juan, PR 00936, USA

**Keywords:** ILK, siRNA, cisplatin, ovarian cancer, RNA-Seq, non-coding RNAs, KM plotter

## Abstract

Despite good responses to first-line treatment with platinum-based combination chemotherapy, most ovarian cancer patients will relapse and eventually develop platinum-resistant disease with poor prognosis. Although reports suggest that integrin-linked kinase (ILK) is a potential target for ovarian cancer treatment, identification of ILK downstream effectors has not been fully explored. The purpose of this study was to investigate the molecular and biological effects of targeting ILK in cisplatin-resistant ovarian cancer. Western blot analysis showed that phosphorylation levels of ILK were higher in cisplatin-resistant compared with cisplatin-sensitive ovarian cancer cells. Further immunohistochemical analysis of ovarian cancer patient samples showed a significant increase in phosphorylated ILK levels in the tumor tissue when compared to normal ovarian epithelium. Targeting ILK by small-interfering RNA (siRNA) treatment reduced cisplatin-resistant cell growth and invasion ability, and increased apoptosis. Differential gene expression analysis by RNA sequencing (RNA-Seq) upon ILK-siRNA transfection followed by Ingenuity Pathway Analysis (IPA) and survival analysis using the Kaplan–Meier plotter database identified multiple target genes involved in cell growth, apoptosis, invasion, and metastasis, including several non-coding RNAs. Taken together, results from this study support ILK as an attractive target for ovarian cancer and provide potential ILK downstream effectors with prognostic and therapeutic value.

## 1. Introduction

Ovarian cancer remains a significant cause of female cancer-related deaths, with approximately 21,750 new cases and 13,940 deaths for 2020 in the US [1]. Its high death rate is reflective of the fact that most ovarian cancer patients are diagnosed with advanced-stage disease [2]. Epithelial ovarian cancer (EOC)—the most common type of ovarian malignancies—is a complex group of diseases that, based on histopathology and molecular genetic alterations, could be divided into five main types including high-grade serous, endometrioid, clear-cell, mucinous, and low-grade serous carcinomas [3,4,5]. High-grade serous ovarian cancer (HGSOC) represents 70% of all EOCs [5]. Despite initial response to first-line treatment with platinum-based combination chemotherapy, most HGSOC patients will relapse and eventually develop platinum-resistant disease with a poor overall prognosis [6]. Therefore, novel therapies targeting key survival pathways are urgently needed to improve clinical outcomes for women with this deadly disease. 

Integrin-linked kinase (ILK) is a highly-conserved serine/threonine kinase and adaptor protein connecting the extracellular matrix (ECM), cell membrane-associated proteins (i.e., integrins), and cell signaling pathways [7,8]. ILK activity is stimulated by integrins and growth factors in a phosphatidylinositol 3-kinase (PI3K)-dependent manner [9]. On the other hand, ILK is negatively regulated by phosphatase and tensin homolog (PTEN) and ILK-associated serine/threonine phosphatase 2C (ILKAP) [9]. In addition, ILK-mediated signaling and intracellular translocation is regulated by p21-activated kinase 1 (PAK1) through ILK phosphorylation at threonine 173 (T173) and serine 246 (S246) residues [10]. Once activated, ILK can mediate a variety of cellular functions through regulation of its downstream targets including protein kinase B (AKT), glycogen synthase kinase 3β (GSK3β), and nuclear factor kappa B (NFκB) [11].

Increased levels of ILK have been associated with cancer progression, epithelial to mesenchymal transition (EMT), angiogenesis, and metastasis [7,8,9,12,13]. In ovarian cancer, ILK expression has been previously associated with tumor progression [14]. Early reports show that subcutaneous injection of ovarian cancer cells transfected with either ILK-short hairpin RNA (shRNA) or ILK-antisense oligonucleotides (ASOs) suppresses tumor formation [15] and growth [16] in vivo. Other studies have shown that ILK activity modulates the pro-metastatic behavior of ovarian cancer [17] by stimulating cell invasion and migration [18]. Moreover, increased ILK activation has been shown to protect platinum-resistant ovarian cancer cells from cisplatin-induced apoptosis [19]. Targeting ILK abrogates the invasive potential of ovarian cancer cells [15], induces apoptosis [20,21], and decreases cell viability [22].

The molecular and biological effects of siRNA-mediated ILK targeting in cisplatin-resistant ovarian cancer and the identification of associated downstream effectors have not been fully explored. In this study, we assessed the phosphorylation levels of ILK in ovarian cancer cell lines and tissue samples and investigated the effects of targeting ILK in cisplatin-resistant ovarian cancer. We report that targeting ILK with siRNA induces significant changes in the expression of several cancer-associated protein-coding genes and non-coding RNAs involved in cell growth, survival, and metastasis. Expression-based survival analysis using the Kaplan–Meier (KM) plotter database revealed significant associations between potential ILK target genes and ovarian cancer prognosis. Results from this study suggest that ILK-regulated genes may serve as potential prognostic markers and therapeutic targets in ovarian cancer.

## 2. Results

### 2.1. Expression Levels of p-ILK and ILK in Ovarian Cancer Cells and Human Ovarian Samples

**Figure 1:**
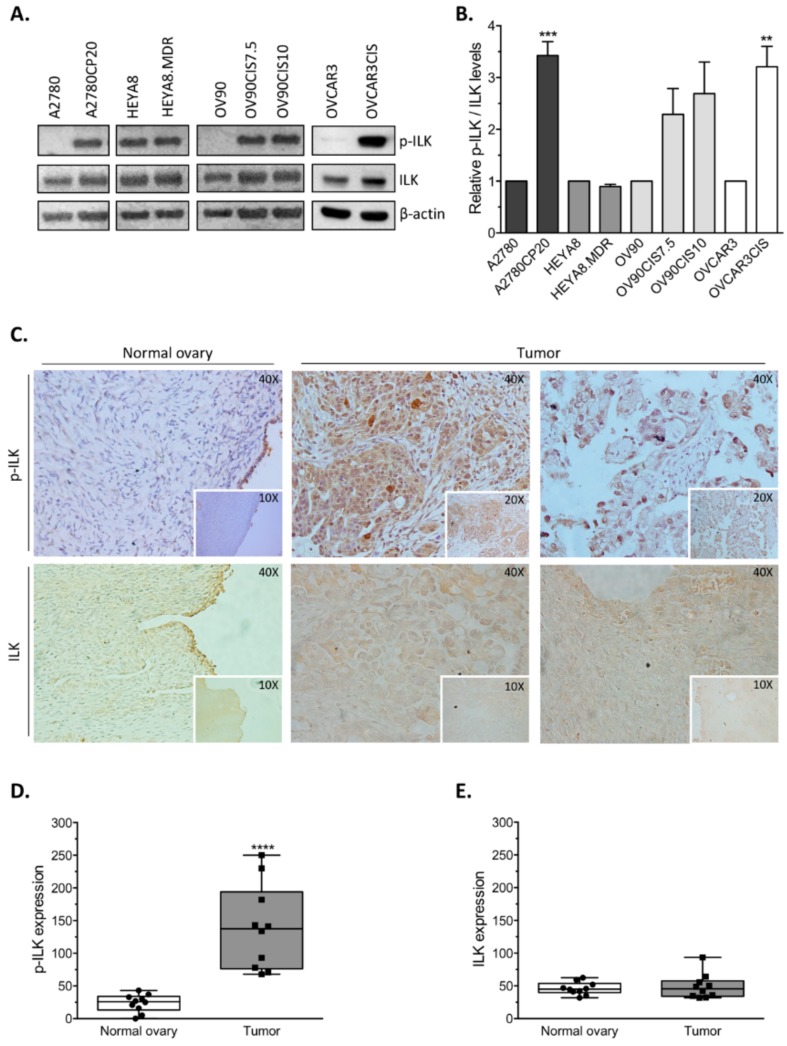
Expression of p-ILK and ILK in ovarian cancer cells and ovarian tumor samples. Western blot analysis and immunohistochemical (IHC) staining were performed. (**A**) Representative Western blots showing the phosphorylated form of ILK (p-ILK) and total ILK protein levels in a panel of ovarian cancer cell lines. (**B)** Densitometric analysis of the band intensities shown in Figure 1A plotted as mean ± SEM (** *p* < 0.01 and *** *p* < 0.001). Phosphorylated ILK/total ILK (p-ILK/ILK) was calculated relative to parental cell lines for each group. (**C**) Representative IHC of p-ILK and ILK protein levels in normal ovary and tumor samples from ovarian cancer patients. (**D**) Expression of p-ILK and (**E**) ILK (positive staining) plotted as mean ±SEM (**** *p* < 0.0001).

Western blot and densitometric analysis of a panel of ovarian cancer cell lines showed that phosphorylation levels of ILK (p-ILK; Ser 246) relative to total ILK protein (p-ILK/ILK) were significantly higher in cisplatin-resistant A2780CP20 and OVCAR3CIS cells compared with their parental counterparts A2780 and OVCAR3, respectively (Figure 1A,B). Although not significant, p-ILK/ILK levels were also higher in cisplatin-resistant OV90CIS cells compared with their parental counterparts OV90 (Figure 1A,B). For taxane-resistant and taxane-sensitive ovarian cancer cells (HEYA8.MDR and HEYA8, respectively) this trend was not observed. We also measured the phosphorylation levels of AKT (p-AKT; Ser 473)—a downstream target of ILK [11]—in cisplatin-resistant ovarian cancer cells and their respective cisplatin-sensitive counterparts. Western blot and densitometric analysis showed that p-AKT levels relative to total AKT protein (p-AKT/AKT) were significantly higher in cisplatin-resistant A2780CP20 and OVCAR3CIS cells compared with their parental counterparts A2780 and OVCAR3, respectively (Figure S1A,B). Although not significant, p-AKT/AKT levels were also higher in cisplatin-resistant OV90CIS cells compared with their parental counterparts OV90 (Figure S1A,B). These observations are consistent with higher p-ILK/ILK protein levels in cisplatin-resistant ovarian cancer cells as compared with their cisplatin-sensitive counterparts (Figure 1A,B).

To assess the clinical significance of p-ILK expression in ovarian cancer, we performed immunohistochemical (IHC) analysis in normal ovary and ovarian cancer tissue sections. Representative IHC images are shown in Figure 1C. A significant increase in p-ILK (**** *p* < 0.0001) was observed in samples from ovarian cancer tissues compared with normal ovary (Figure 1D). No significant change was observed for total ILK (Figure 1C,E).

### 2.2. Effects of ILK-siRNA Transfection on Cell Growth, Invasion, and Viability

**Figure 2:**
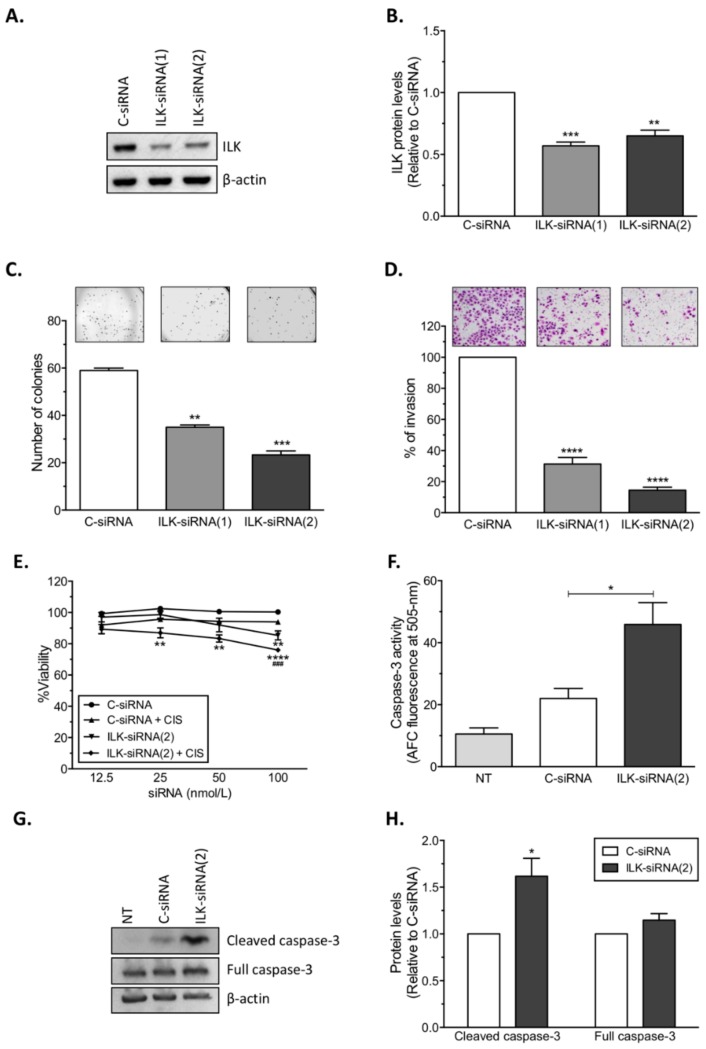
SiRNA-mediated ILK targeting in A2780CP20 cells. SiRNAs were transiently transfected into ovarian cancer cells. A reduction in (**A**,**B**) ILK protein levels, (**C**) colony formation, (**D**) invasion ability, and (**E**) cell viability was observed following ILK-siRNA transfection into A2780CP20 cells. Attached and floating cells were collected 72 h after transfection. (**F**) Protein extracts from siRNA-transfected cells were used to assess caspase-3 activity (DEVD-AFC cleavage). Increased caspase-3 activity was observed upon ILK-siRNA transfection. (**G**) Representative Western blot showing full caspase-3 and its cleavage product. (**H**) Densitometric analysis of the band intensities shown in Figure 2G. Mean ± SEM is shown relative to C-siRNA (* *p* < 0.05, ** *p* < 0.01, *** *p* < 0.001, and **** *p* < 0.0001) or C-siRNA + CIS (### *p* < 0.001).

Next, we studied whether targeting ILK reduces cell growth and the invasive ability of cisplatin-resistant ovarian cancer cells. Transient transfection of ILK-targeted siRNAs into A2780CP20 cells decreased ILK protein levels (43.0% reduction; *** *p* < 0.001 and 35.0% reduction; ** *p* < 0.01) compared with C-siRNA-transfected cells (Figure 2A,B). In a colony formation assay with A2780CP20, both ILK-targeted siRNAs reduced the number of colonies formed compared with C-siRNA-transfected cells (Figure 2C). Particularly, ILK-siRNA(2) reduced in 60.5% (*** *p* < 0.001) the number of colonies, whereas ILK-siRNA(1) reduced the number of colonies in only 40.7% (** *p* < 0.01). Invasion assays confirmed that ILK-siRNA(1) and ILK-siRNA(2) significantly reduced (68.7% reduction; **** *p* < 0.0001 and 85.5% reduction; **** *p* < 0.0001, respectively) the invasiveness of A2780CP20 cells compared with C-siRNA-transfected cells (Figure 2D). 

Then, we investigated whether ILK-targeted siRNAs alone or in combination with cisplatin (CIS) induced effects in cell viability. Transient transfection of 100 nmol/L of ILK-siRNA(2) into A2780CP20 cells significantly reduced (** *p* < 0.01) cell viability compared with C-siRNA (Figure 2E). Combination of ILK-siRNA(2) with CIS (2 µmol/L) significantly reduced (** *p* < 0.01) cell viability at siRNA doses as low as 25 nmol/L compared with C-siRNA (Figure 2E). To assess whether the effects induced on cell viability by ILK-siRNA were due to apoptosis, activation of caspase-3 was measured in cisplatin-resistant ovarian cancer cells. A significant increase (* *p* < 0.05) in caspase-3 activity was observed after transient transfection of 100 nmol/L of ILK-siRNA(2) into A2780CP20 cells compared with C-siRNA-transfected cells (Figure 2F). Results were validated by Western blot analysis, which showed increased caspase-3 cleavage upon ILK targeting (Figure 2G,H).

**Figure 3:**
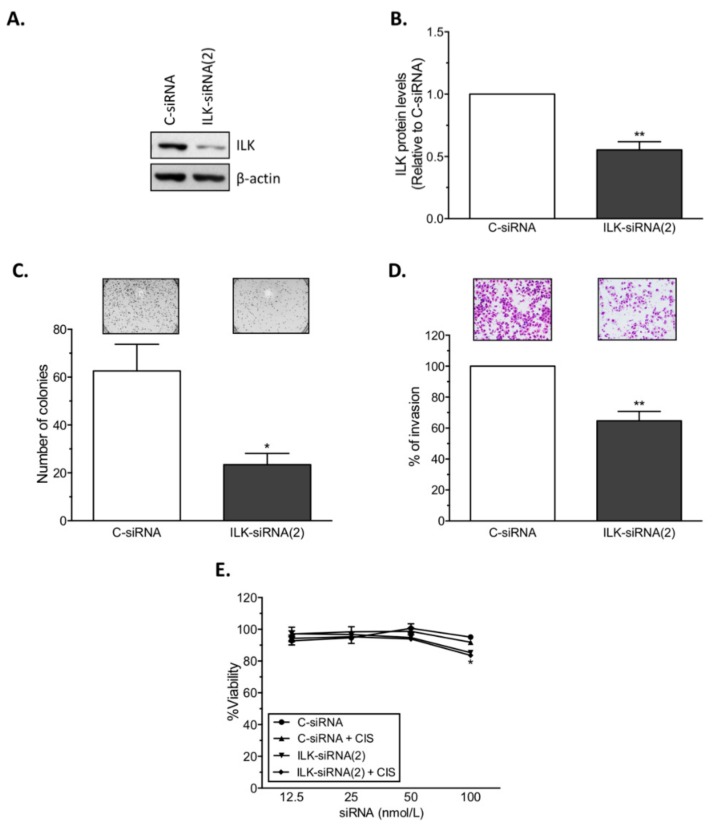
SiRNA-mediated ILK targeting in OVCAR3CIS cells. SiRNAs were transiently transfected into ovarian cancer cells. A reduction in (**A**,**B**) ILK protein levels, (**C**) colony formation, (**D**) invasion ability, and (**E**) cell viability was observed following ILK-siRNA transfection into OVCAR3CIS cells. Mean ± SEM is shown relative to C-siRNA (* *p* < 0.05 and ** *p* < 0.01).

Similar to A2780CP20, transient transfection of ILK-siRNA(2) into OVCAR3CIS cells—a HGSOC cell line resistant to CIS—reduced ILK protein levels (45.0% reduction; ** *p* < 0.01, Figure 3A,B), colony formation (62.5% reduction; * *p* < 0.05, Figure 3C), and cell invasion (35.3% reduction; ** *p* < 0.01, Figure 3D) compared with C-siRNA-transfected cells. Combination of ILK-siRNA(2) with CIS (2 µmol/L) significantly reduced (* *p* < 0.05) cell viability at 100 nmol/L compared with C-siRNA (Figure 3E). 

Similar results for ILK protein levels (37.0% reduction; ** *p* < 0.01, Figure S2A,B), colony formation (66.8% reduction; * *p* < 0.05, Figure S2C), and cell invasion (61.6% reduction; **** *p* < 0.0001, Figure S2D) were observed when transfecting ILK-siRNA(2) into OV90CIS cells, another HGSOC cell line resistant to CIS. In addition, transient transfection of ILK-siRNA(2) into HEYA8 cells decreased ILK protein levels compared with C-siRNA-transfected cells (44.0% reduction; *** *p* < 0.001, Figure S2E,F). However, in contrast to A2780CP20 and OVCAR3CIS cells, ILK-siRNA(2) significantly reduced (** *p* < 0.01) the viability of HEYA8 cells at concentrations of 25 nmol/L or lower compared with C-siRNA (Figure S2G). Importantly, no significant changes in colony formation and/or viability were observed when ILK-siRNA(2) was transfected into A2780, OVCAR3, and OV90 cells (Figure S3A–E). 

### 2.3. Effect of a Small-Molecule ILK Inhibitor on Cell Viability

Next, we studied the effect of a small-molecule ILK inhibitor (Cpd22) on cell viability. Inhibition of ILK activity by Cpd22 has been shown to suppress the viability of prostate and breast cancer cells [23]. Treatment with Cpd22 significantly reduced the viability of both cisplatin-resistant (A2780CP20, OVCAR3CIS, and OV90CIS) and cisplatin-sensitive (A2780, OVCAR3, and OV90) ovarian cancer cells in a dose-dependent manner (Figure S4A–F). The viability of HEYA8 cells was also significantly reduced by Cpd22 treatment (Figure S4G). Western blot and densitometric analysis showed that p-ILK levels relative to total ILK protein (p-ILK/ILK) were significantly lower in OVCAR3CIS (25.3% reduction; ** *p* < 0.01, Figure S5A,B) and HEYA8 cells (20.7% reduction; ** *p* < 0.01, Figure S5C,D) upon Cpd22 (2 µmol/L) treatment. Although not significant, a reduction in p-ILK/ILK levels was also observed for A2780CP20 (26.7% reduction; p = n.s., Figure S5E,F) and OV90CIS cells (14% reduction; p = n.s., Figure S5G,H).

### 2.4. Effect of ILK-siRNA Transfection on Gene Expression

To further assess the signaling pathways altered as a result of ILK-siRNA transfection in cisplatin-resistant ovarian cancer cells, we performed a transcriptome-wide analysis by RNA sequencing (RNA-Seq). Using an initial false discovery rate (FDR, q-value) cutoff of <0.05, we identified 2028 differentially expressed genes between C-siRNA and ILK-siRNA-transfected cells including 1322 upregulated and 706 downregulated genes (data not shown). 

Differentially expressed genes with a log2 fold change of ≥1.5 and ≤−1.5 were used to analyze functional enrichment using the Kyoto Encyclopedia of Genes and Genomes (KEGG). The top 10 most significantly enriched KEGG pathways include metabolic pathways, olfactory transduction, pathways in cancer, PI3K-AKT signaling pathway, MAPK signaling pathway, neuroactive ligand–receptor interaction, HTLV-I infection, cytokine–cytokine receptor interaction, proteoglycans in cancer, and focal adhesion (Table 1).

Gene ontology (GO) analysis of significantly enriched processes, components, and functions with a FDR < 0.01 showed that differentially expressed genes were mainly associated with tissue development, positive regulation of macromolecule biosynthetic process, cell development, positive regulation of RNA metabolic process, and regulation of multicellular organismal development within the biological processes category (Table 2); adenyl nucleotide binding, adenyl ribonucleotide binding, ATP binding, signal transducer activity, and transition metal ion binding within the molecular functions category (Table 2); and intrinsic component of plasma membrane, integral component of plasma membrane, neuron part, Golgi apparatus, and cell junction within the cellular components category (Table 2). 

A dataset containing the gene symbols, fold change (log2), and *p*-values of the 2028 differentially expressed genes was uploaded into the Ingenuity Pathway Analysis (IPA) software. Of these genes, 1804 were mapped successfully by IPA. To select differentially expressed genes, a log2 fold change cutoff >1.5 or <−1.5 with a *p*-value ≤ 0.001 was used. Using these criteria, we identified a total of 77 differentially expressed genes, 68 upregulated and 9 downregulated in ILK-siRNA vs. C-siRNA-transfected cells (Table S1). Based on fold change, among the 68 upregulated genes, the top 5 include *GRIA4*, *SCG3*, *CHRNB2*, *XKR7*, and *TOMM40L* (Table 3). Among the nine downregulated genes, the top five include *NSG1*, *TEX41*, *SLC4A8*, *SAG*, and *ILK* (Table 3).

In agreement with evidence that long non-coding RNAs (lncRNAs) are key regulators of gene expression [24], using an FDR < 0.05, we identified 296 lncRNAs with altered expression following ILK-siRNA transfection, 205 upregulated and 91 downregulated (data not shown). To select differentially expressed lncRNAs, a log2 fold change cutoff >1.0 or <−1.0 with a *p*-value ≤ 0.001 was used. Using these criteria, we identified a total of 37 differentially expressed lncRNAs, 33 upregulated and 4 downregulated (Table S2). Based on fold change, the top 10 lncRNAs include *MIR7-3HG*, *LINC01134*, *RP11-64K12.4*, *RP11-799D4.4*, *RP11-618K13.2*, *RP11-380L11.4*, *ARHGEF26-AS1*, *RP11-732A19.8*, *RP11-20G6.3*, and *RP11-474G23.2* (Table 4). 

### 2.5. IPA Analysis of Differentially Expressed Genes

To better examine the interaction networks of ILK downstream genes, we performed IPA analysis with the 77 differentially expressed transcripts. This analysis produced a list of 9 significantly altered canonical pathways (*p* < 0.05 or –log *p*-value > 1.30) including SAPK/JNK signaling, NAD biosynthesis and salvage pathway, phototransduction pathway, VDR/RXR activation, Reelin signaling in neurons, and role of IL-17A in psoriasis (Table 5). 

**Figure 4:**
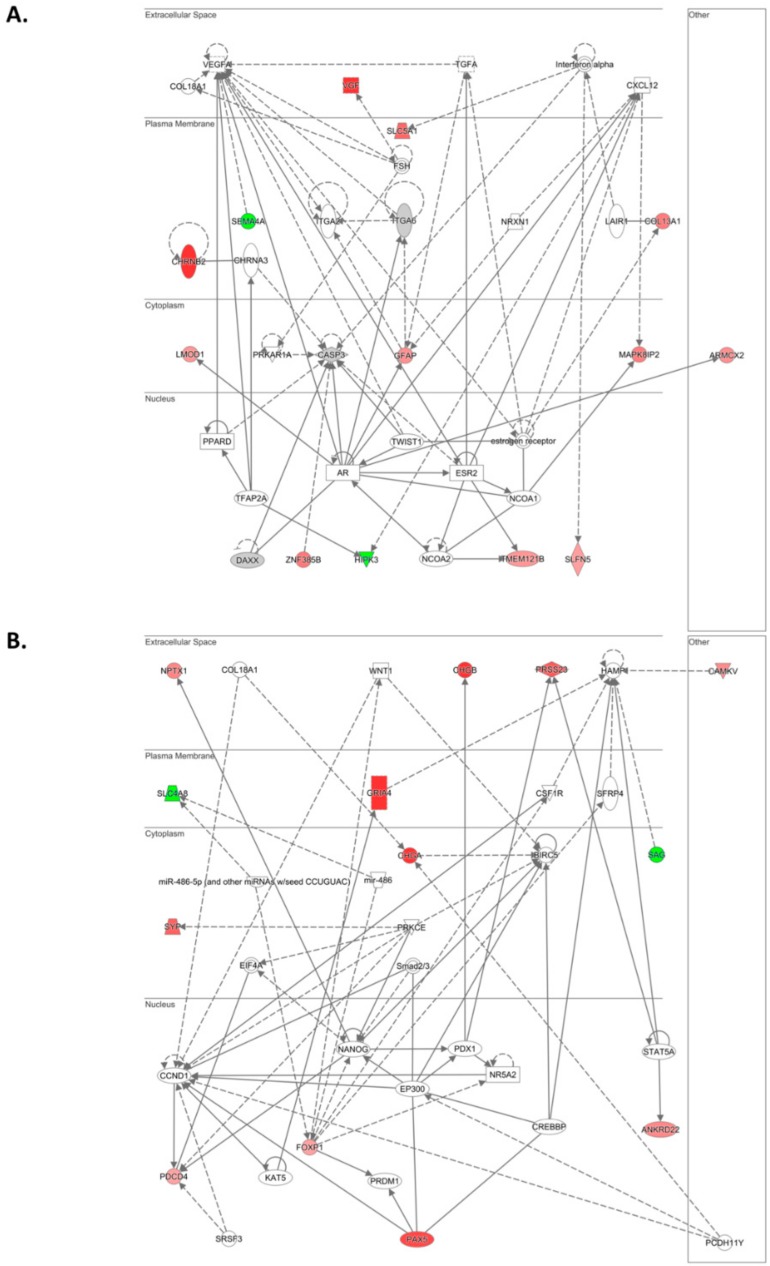
Network analysis by Ingenuity Pathway Analysis (IPA). The top two altered networks following siRNA-mediated ILK targeting are shown. (**A**) The top network includes molecules associated with nervous system development and function, organ morphology, and organismal development. (**B**) The second top network includes molecules associated with cell death and survival, cancer, and immunological disease. Green and red symbols denote downregulated and upregulated genes, respectively.

The top network with an IPA score of 23 and composed of 13 molecules was associated with nervous system development and function, organ morphology, and organismal development (Figure 4A and Table S3). The second top network (score: 23, molecules: 13) was associated with cell death and survival, cancer, and immunological disease (Figure 4B and Table S3). 

In addition, other networks within the top 5 list were associated with cellular movement, cell-to-cell signaling and interaction, cellular development, and cellular growth and proliferation (Table S3). Furthermore, cell morphology, cellular assembly and organization, and cellular function and maintenance were within the top 5 cellular and molecular functions associated with ILK downregulation (Table 6).

### 2.6. Prognostic Value of ILK Downstream Genes

**Figure 5:**
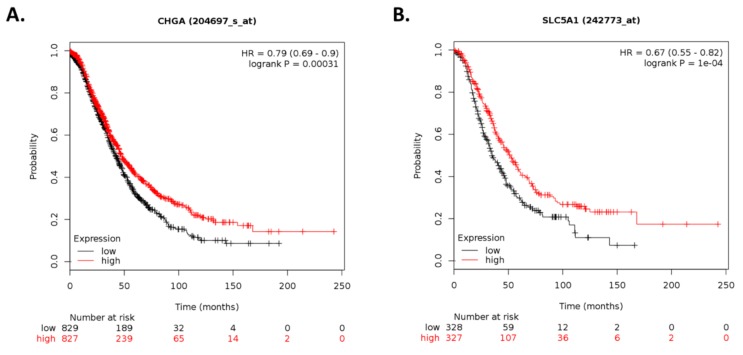

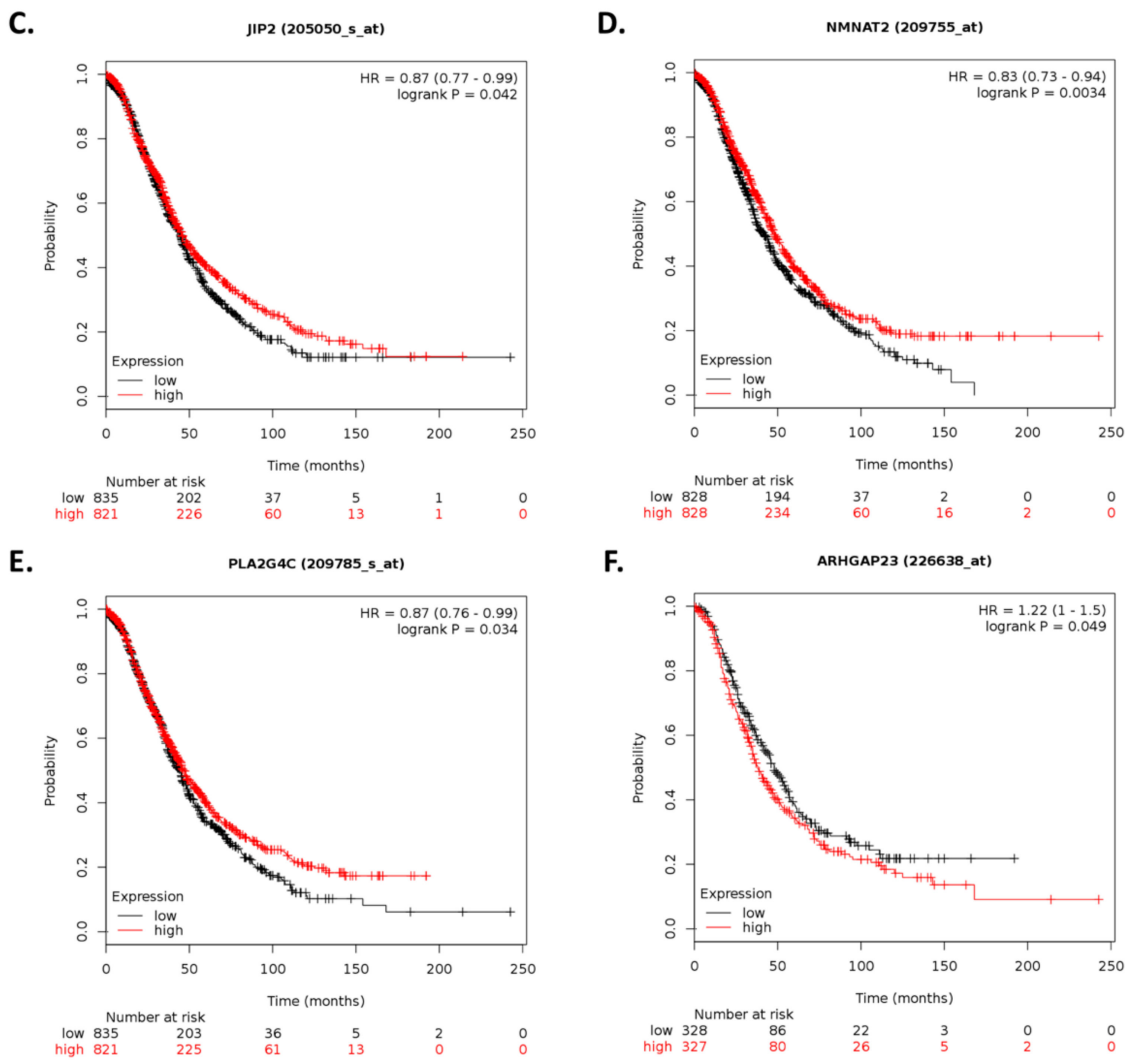
Kaplan–Meier plots for gene expression-based overall survival analysis. Survival plots of ovarian cancer patients were generated using Kaplan–Meier plotter (KM plotter). Overall survival (OS) of patients with ovarian cancer stratified by expression levels of (**A**) *CHGA*, (**B**) *SLC5A1*, (**C**) *MAPK8IP2*, (**D**) *NMNAT2*, (**E**) *PLA2G4C*, and (**F**) *ARHGAP23* is shown based on gene chip data. *p*-values < 0.05 were considered to be statistically significant.

To assess whether the differentially expressed transcripts identified upon ILK targeting are clinically relevant in ovarian cancer, we conducted survival analysis using Kaplan–Meier plotter (KM plotter) [25]. Gene chip data (Affymetrix) for 70 out of the 77 differentially abundant transcripts (Table S1) were available in the database. After dividing the patients into high and low expression (according to the median value), 33 of the 70 transcripts were significantly associated with overall survival (OS) and/or progression-free survival (PFS; Table S4). Expression levels upon ILK depletion for 10 out of the 33 transcripts were concordant with survival outcomes (Table S4). A significant association between high expression levels and better prognosis (OS; HR < 1) was found for *CHGA*, *SLC5A1*, *MAPK8IP2*, *NMNAT2*, and *PLA2G4C*, whereas worse outcomes (OS; HR > 1) were associated with high expression of *ARHGAP23* (Figure 5).

**Figure 6:**
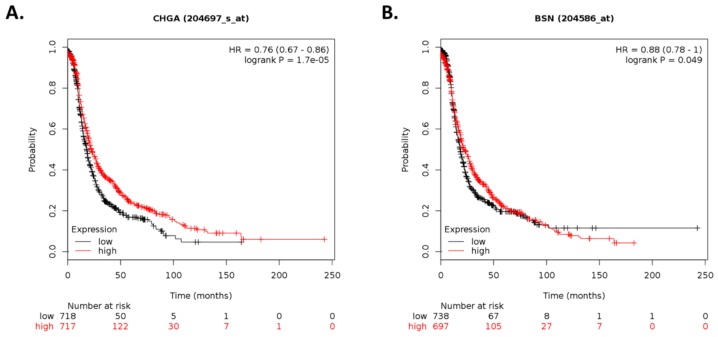

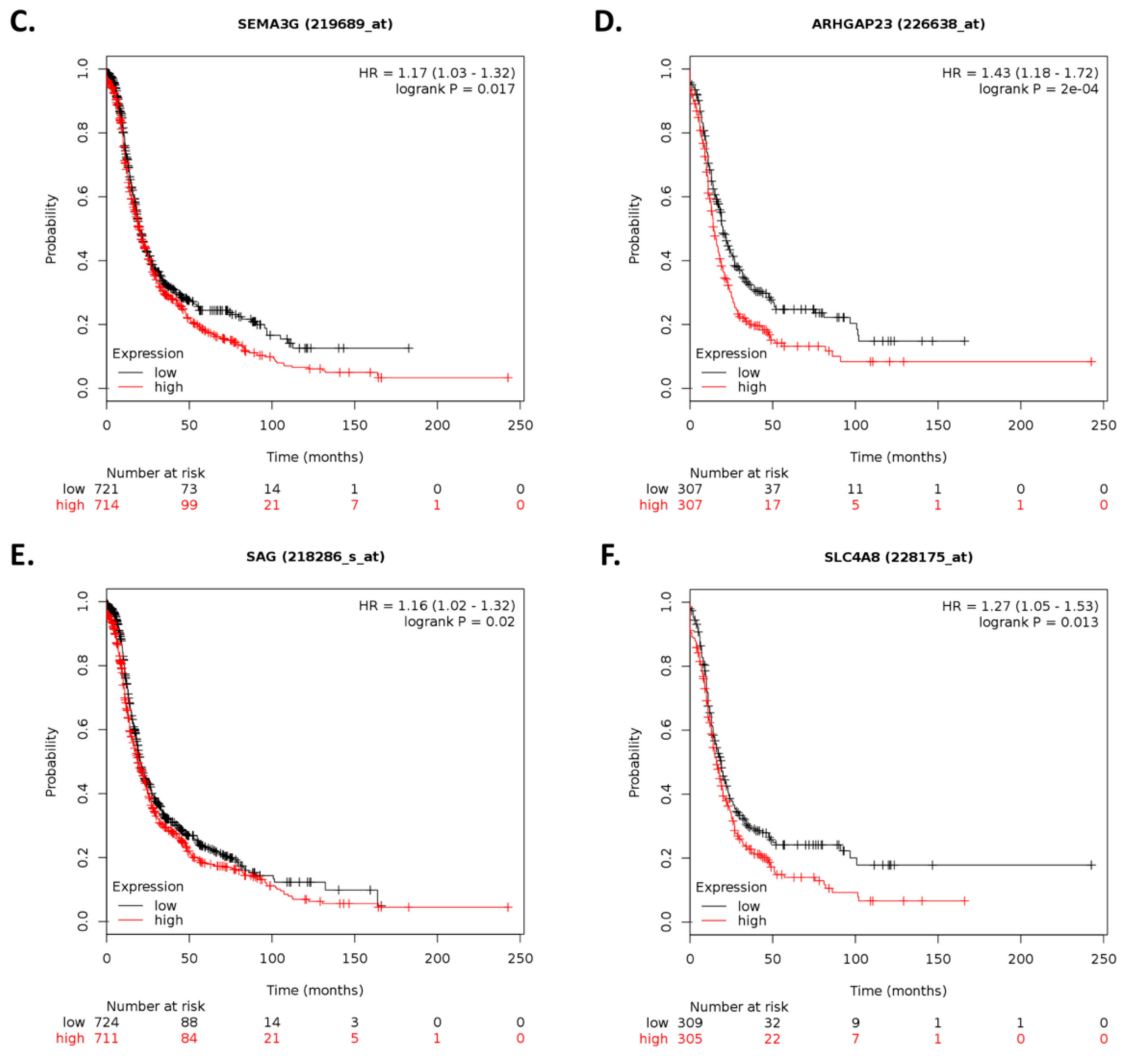
Kaplan–Meier plots for gene expression-based progression-free survival analysis. Survival plots of ovarian cancer patients were generated using Kaplan–Meier plotter (KM plotter). Progression-free survival (PFS) of patients with ovarian cancer stratified by expression levels of (**A**) *CHGA*, (**B**) *BSN*, (**C**) *SEMA3G*, (**D**) *ARHGAP23*, (**E**) *SAG*, and (**F**) *SLC4A8* are shown based on gene chip data. *p*-values < 0.05 were considered to be statistically significant.

In addition, high expression levels of *CHGA* and *BSN* were associated with longer PFS (HR < 1), whereas high expression of *SEMA3G*, *ARHGAP23*, *SAG*, and *SLC4A8* were associated with shorter PFS (HR > 1; Figure 6). 

For ovarian cancer patients treated with platin, 30 of the 70 transcripts were significantly associated with OS and/or PFS (Table S5). Expression levels upon ILK depletion for 11 out of the 30 transcripts were concordant with survival outcomes (Table S5). A significant association between high expression levels and better prognosis (OS; HR < 1) was found for *VGF*, *CHGA*, and *NMNAT2*, whereas worse outcomes (OS; HR > 1) were associated with high expression of *ARHGAP23* (Figure S6). In addition, high expression levels of *CHGA*, *ACTL6B*, *BSN*, *PAX5*, *NKAIN1*, *SYP*, *CAMKV* were associated with longer PFS (HR < 1), whereas high expression of *ARHGAP23* and *SLC4A8* were associated with shorter PFS (HR > 1; Figure S7).

For serous ovarian cancer patients, 27 of the 70 transcripts were significantly associated with OS and/or PFS (Table S6). Expression levels upon ILK depletion for 7 out of the 27 transcripts were concordant with survival outcomes (Table S6). A significant association between high expression levels and better prognosis (OS; HR < 1) was found for *CHGA*, *SLC5A1*, and *NMNAT2*, whereas worse outcomes (OS; HR > 1) were associated with high expression of *ARHGAP23* (Figure S8). In addition, high expression levels of *SEMA3G*, *ARHGAP23*, *ILK*, and *SAG* were associated with shorter PFS (HR > 1; Figure S9).

For serous ovarian cancer patients treated with platin, 23 of the 70 transcripts were significantly associated with OS and/or PFS (Table S7). Expression levels upon ILK depletion for 5 out of the 23 transcripts were concordant with survival outcomes (Table S7). A significant association between high expression levels and better prognosis (OS; HR < 1) was found for *LTF*, whereas worse outcomes (OS; HR > 1) were associated with high expression of *ARHGAP23* (Figure S10). In addition, high expression levels of *SEMA3G*, *ARHGAP23*, *ILK*, and *SLC4A8* were associated with shorter PFS (HR > 1; Figure S11).

**Figure 7:**
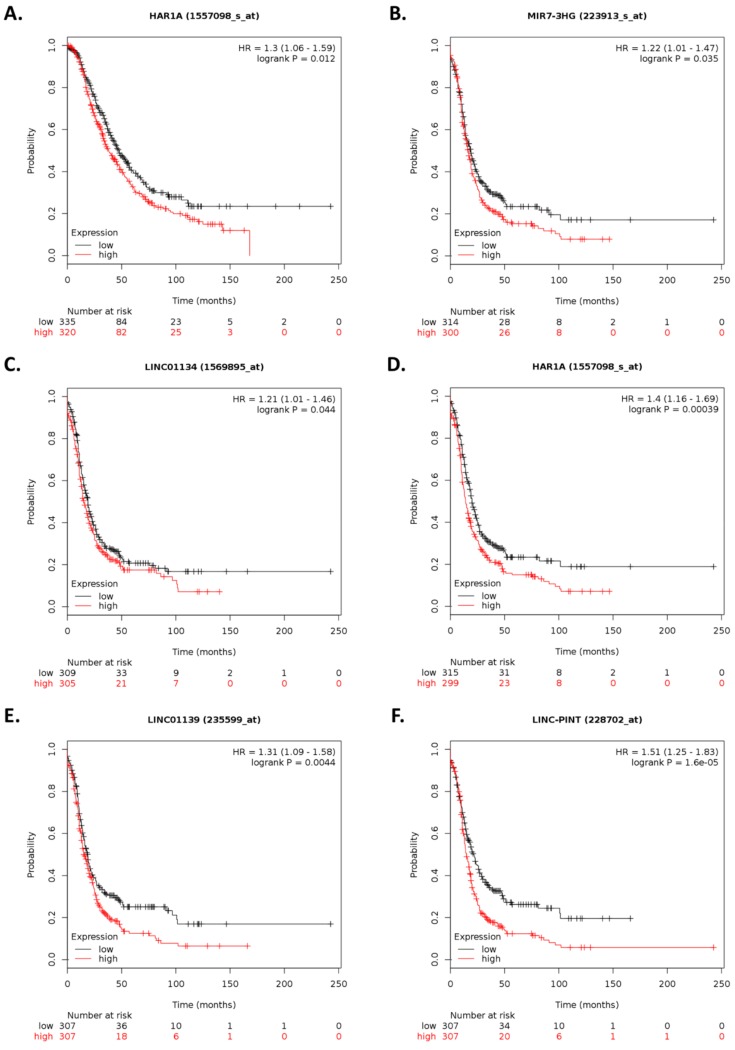

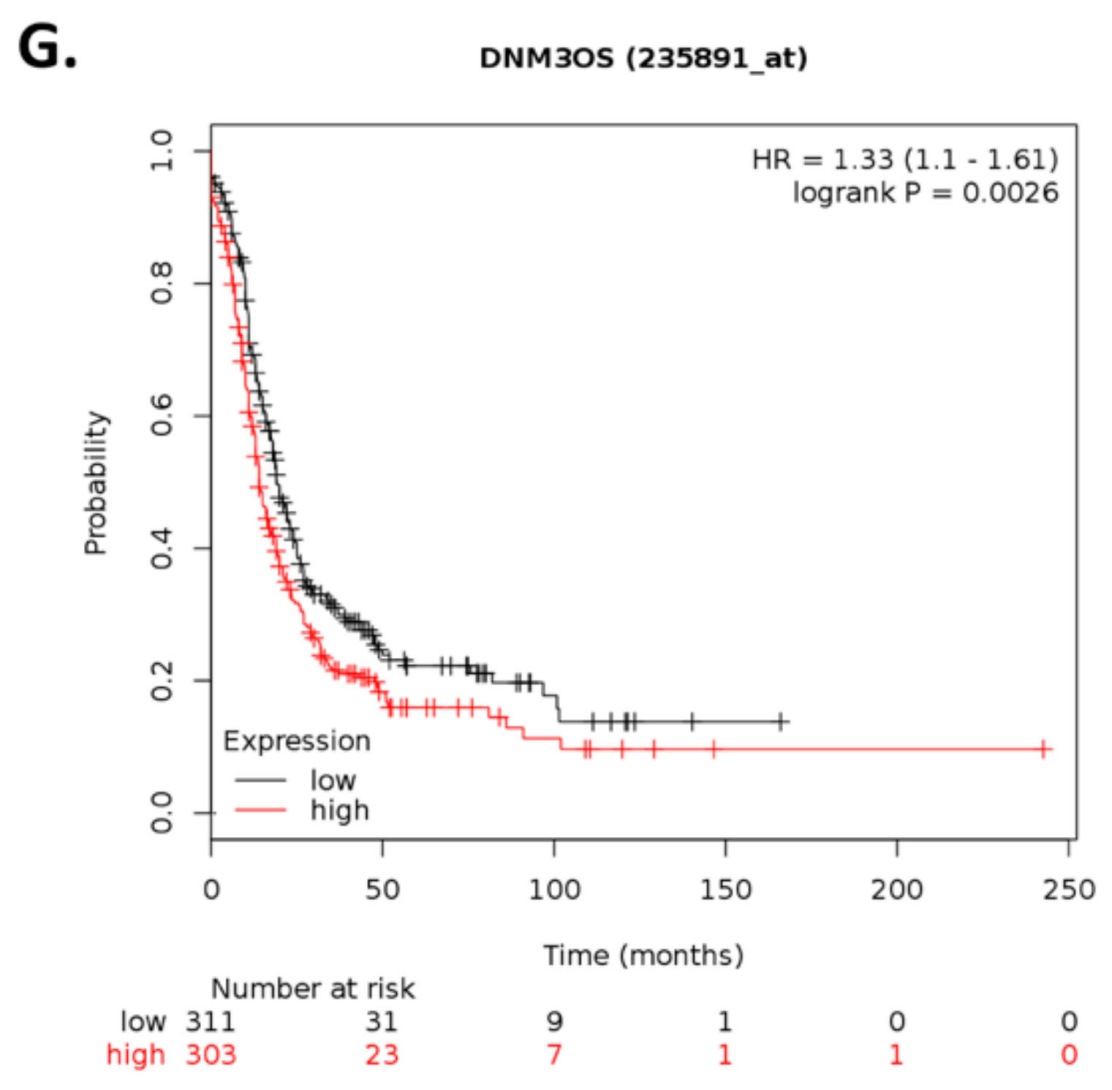
Kaplan–Meier plots for lncRNA expression-based overall survival and progression-free survival analysis. Survival plots of ovarian cancer patients were generated using Kaplan–Meier plotter (KM plotter). Overall survival (OS) of patients with ovarian cancer stratified by expression levels of (**A**) *HAR1A* is shown based on gene chip data. Progression-free survival (PFS) of patients with ovarian cancer stratified by expression levels of (**B**) *MIR7-3HG*, (**C**) *LINC01134*, (**D**) *HAR1A*, (**E**) *LINC01139*, (**F**) *LINC-PINT*, and (**G**) *DNM3OS* are shown based on gene chip data. *p*-values < 0.05 were considered to be statistically significant.

Gene chip data for 11 of the 37 differentially abundant lncRNAs following ILK targeting (Table S2) were also available in KM plotter. After dividing the patients into high and low expression (according to the median value), 6 of 11 lncRNAs including *MIR7-3HG*, *LINC01134*, *HAR1A*, *LINC01139*, *LINC-PINT*, and *DNM3OS* were significantly associated with OS and/or PFS (Figure 7 and Table S8). However, expression levels of these lncRNAs upon ILK depletion were not concordant with survival outcomes. 

For ovarian cancer patients treated with platin, five lncRNAs including *LINC01134*, *HAR1A*, *LINC01139*, *LINC-PINT*, and *DNM3OS* were significantly associated with PFS (Figure S12 and Table S9). For serous ovarian cancer patients, three lncRNAs including *HAR1A*, *LINC00886*, and *LINC-PINT* were significantly associated with PFS (Figure S13 and Table S10). For serous ovarian cancer patients treated with platin, five lncRNAs including *HAR1A*, *LINC00886*, *LINC01139*, *LINC-PINT*, *DNM3OS* were significantly associated with PFS (Figure S14 and Table S11). However, expression levels of these lncRNAs upon ILK depletion were not concordant with survival outcomes.

**Figure 8:**
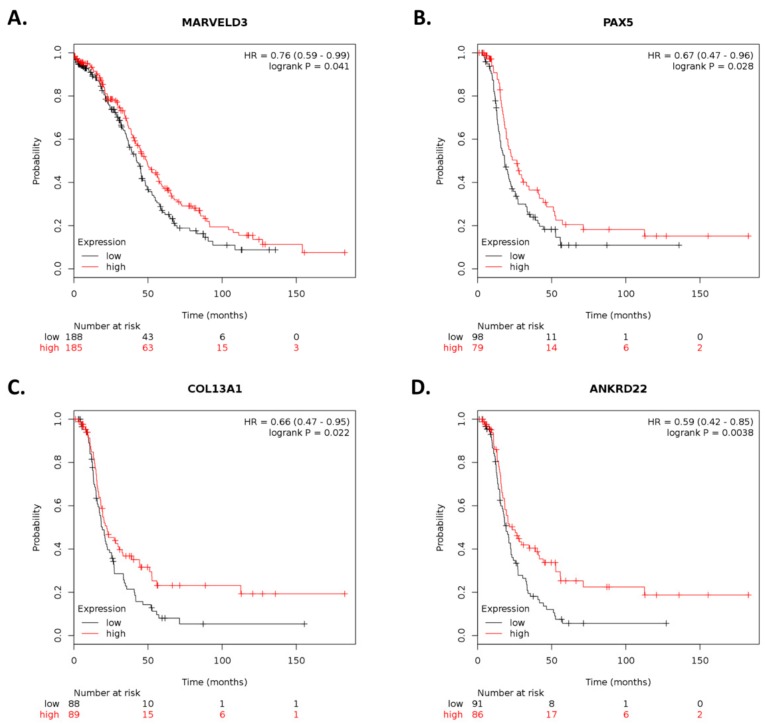
Kaplan–Meier plots for gene expression-based overall survival and relapse-free survival analysis. Survival plots of ovarian cancer patients were generated using Kaplan–Meier plotter (KM plotter). Overall survival (OS) of patients with ovarian cancer stratified by expression levels of (**A**) *MARVELD3* is shown based on RNA-Seq data. Relapse-free survival (RFS) of patients with ovarian cancer stratified by expression levels of (**B**) *PAX5*, (**C**) *COL13A1*, and (**D**) *ANKRD22* is shown based on RNA-Seq data. *p*-values < 0.05 were considered to be statistically significant.

Survival analysis was also conducted using Pan-cancer RNA-Seq data in KM plotter. Expression levels for 75 of the 77 differentially abundant transcripts (Table S1) were available in the database. After dividing ovarian cancer patients into high and low expression (according to the median value), 9 of 75 transcripts (Table S12) were found significantly associated with OS (*n* = 373) or relapse-free survival (RFS; *n* = 177). Expression levels of four of the nine transcripts were concordant with survival outcomes (Table S12). High expression levels of *MARVELD3* were associated with longer OS (HR < 1), whereas high expression levels of *PAX5*, *COL13A1*, and *ANKRD22* were associated with longer RFS (HR < 1; Figure 8). Although expression levels for 10 of the 37 differentially abundant lncRNAs were available from RNA-Seq data in KM plotter, no significant associations were found between lncRNA expression and OS or RFS. 

### 2.7. Target Validation by qRT-PCR Analysis

Based on RNA-Seq data upon ILK targeting in cisplatin-resistant ovarian cancer cells and survival analysis of differentially expressed genes, *ACTL6B*, *ANKRD22*, *ARHGAP23*, *BSN*, *CAMKV*, *CHGA*, *COL13A1*, *ILK*, *LTF*, *MAPK8IP2*, *MARVELD3*, *NKAIN1*, *NMNAT2*, *PAX5*, *SAG*, *SEMA3G*, *SLC4A8*, *SLC5A1*, and *VGF* were selected for further validation by SYBR Green-based qRT-PCR analysis. Expression levels of all genes tested were concordant with RNA-Seq data except for *NKAIN1* (Table 7). *ARHGAP23* and *COL13A1* did not show significant differences in expression (Table 7). Expression of *LTF* and *SAG* was not detected by qRT-PCR.

## 3. Discussion

Increased levels of ILK, a serine/threonine kinase and adaptor protein, have been well documented in different tumor types, including ovarian cancer [14]. Here, we present further evidence that phosphorylated ILK (p-ILK) levels are higher in human ovarian cancer tissues compared with normal ovary samples, and that p-ILK levels relative to total ILK protein (p-ILK/ILK) are higher in cisplatin-resistant compared with cisplatin-sensitive ovarian cancer cells. Transfection of ILK-siRNA into cisplatin-resistant ovarian cancer cells induced long-term effects on cell growth, short-term effects on cell viability in combination with cisplatin treatment, and increased caspase-3 activity. These results are in accordance with previous reports showing that targeting ILK decreases cell viability [22] and induces apoptosis in ovarian cancer [20,21], and increases the sensitivity of lung cancer [26], gastric carcinoma [27], and colon cancer [28] cells to cisplatin or oxaliplatin treatment. In fact, studies in platinum-resistant ovarian cancer have shown that ILKAP, a phosphatase which inactivates ILK, is downregulated by cisplatin treatment, and that silencing *ILKAP* increases ILK activation and protects cells from cisplatin-induced apoptosis [19]. We also observed that transfection of ILK-siRNA reduced the invasive potential of cisplatin-resistant ovarian cancer cells, which is in agreement with previous studies showing that ILK activity modulates the pro-metastatic behavior of ovarian cancer [17]. Similar to our results, ILK activity stimulates invasion and migration of the SKOV3 human ovarian cancer cell line [18], whereas ILK depletion by shRNA (short hairpin RNA) abrogates the invasive potential of SKOV3 cells [15]. 

Inhibition of ILK activity by Cpd22 treatment significantly reduced the viability of all cancer cell lines tested. These results are in accordance with a previous study showing that Cpd22 treatment suppresses the viability of prostate and breast cancer cells compared with normal prostate and mammary epithelial cells [23]. Cpd22 inhibits ILK kinase activity, facilitating dephosphorylation of ILK targets, suppressing the expression of YB-1 (Y-box binding protein) transcription factor and its targets, and inducing autophagy and apoptosis [23]. In addition, ILK expression levels positively correlate with the efficacy of Cpd22 in leukemia [29]. While siRNAs have high specificity in a sequence specific manner, small-molecule inhibitors can act over multiple targets [30]. These observations could explain in part the fact that the viability of both cisplatin-resistant and cisplatin-sensitive cells was reduced upon Cpd22 treatment.

Results from RNA-Seq analysis of ILK-siRNA-transfected ovarian cancer cells showed significant changes in the expression of cancer-associated molecules including protein-coding genes and lncRNAs. Repression of glutamate receptor *GRIA4*, one of the top upregulated genes in our study, increases cell viability, proliferation, and migratory potential in rhabdomyosarcoma/medulloblastoma and multiple myeloma cells [31]. Another *GRIA* family member, *GRIA2*, is downregulated in chemoresistant advanced serous papillary ovarian carcinomas and upregulated in chemosensitive tumors [32]. *TOMM40L*, also upregulated in our study, is upregulated in epithelial ovarian cancer cell lines overexpressing DOK1, a candidate tumor suppressor associated with cisplatin sensitivity [33]. Similarly, *TOMM40L* is downregulated in gemcitabine-resistant pancreatic cancer cells compared with their parental sensitive counterparts [34]. Inhibition of NDCBE in cholangiocarcinoma cells, a product of the *SLC4A8* gene downregulated in our study, decreases proliferation and induces apoptosis [35]. *TEX41*, also downregulated in our study, is upregulated in portal vein tumor thrombosis (PVTT), the main route for intrahepatic metastasis in hepatocellular carcinoma [36]. Therefore, changes in the expression of these genes following siRNA-mediated ILK depletion in cisplatin-resistant ovarian cancer cells could explain in part the reduction in cell growth, invasion, and viability, and the increase in caspase-3 activity.

In agreement with these observations, within the list of differentially abundant transcripts, we identified additional genes involved in the regulation of cell proliferation, survival, migration, invasion, metastasis, drug resistance, and apoptosis. Interestingly, *DUSP8*, *MARVELD3*, *PDCD4*, *MAPK8IP1*, *MAPK8IP2*, and *HIPK3* converge on JNK signaling, one of the top canonical pathways (SAPK/JNK) identified in our IPA analysis. In epithelial ovarian cancer, activated JNK is associated with decreased PFS [37]. In addition, we have previously shown that the JNK-1/c-JUN/*miR-21* pathway contributes to cisplatin resistance in ovarian cancer cells [38]. ILK negatively regulates the expression of *DUSP8* [39], a MAPK phosphatase upregulated in our study, and DUSP8 dephosphorylates and inactivates JNK [40,41]. Inhibition of ILK/AKT decreases *miR-21* in vestibular schwannoma and meningioma cells [42], and targeting *miR-21* with antisense oligonucleotides (ASOs) inhibits growth and metastasis via upregulation of DUSP8 in colorectal carcinoma [43]. MARVELD3, a transmembrane protein of tight junctions [44,45] upregulated in our study, inhibits JNK activity via recruitment of MEKK1 to cell junctions [46]. MARVELD3 is downregulated in SNAIL-induced EMT during pancreatic cancer progression [47]. Similarly, *MARVELD3* silencing decreases *CDH1* and increases *SNAI2* expression in lung cancer [48]. Downregulation of MARVELD3 in colorectal cancer cells increases migration and proliferation, whereas upregulation inhibits migration, proliferation, and in vivo tumor formation [46]. PDCD4, a well-known tumor suppressor [49] upregulated in our study, has been suggested to interact with c-JUN, blocking c-JUN phosphorylation by JNK [50]. In ovarian cancer, lower PDCD4 expression correlates with shorter disease-free survival (DFS) [51]. Moreover, PDCD4 overexpression enhances the sensitivity of ovarian cancer cells to cisplatin by activating the death receptor pathway [52]. In bladder cancer cells, PDCD4 overexpression enhances sensitivity to cisplatin via regulation of the JNK/c-JUN pathway [53]. The JIP family of MAPK scaffold proteins, including MAPK8IP1 and MAPK8IP2 which were upregulated in our study, have been previously identified as regulators of JNK signaling [54]. MAPK8IP1, a negative regulator of MAPK activity [55], inhibits c-JUN phosphorylation by JNK [56]. In gastric cancer, *miR-10a* promotes cell migration and invasion by downregulating *MAPK8IP1* [55]. HIPK3, a FAS/FADD-interacting kinase [57] downregulated in our study, has been previously implicated in multidrug resistance in cancer [58,59]. In fact, overexpression of *HIPK3* protects osteosarcoma cells from cisplatin-induced death in vitro and in vivo [60]. In prostate cancer, JNK activity increases the expression of HIPK3 and promotes resistance to FAS receptor-mediated apoptosis [61]. Overall, these observations suggest that ILK regulates the JNK/c-JUN pathway in cisplatin-resistant ovarian cancer via modulation of *DUSP8*, *MARVELD3*, *PDCD4*, *MAPK8IP1*, *MAPK8IP2*, and *HIPK3*.

Within the top 10 differentially expressed lncRNAs, *ARHGEF26-AS1* and *MIR7-3HG* have been previously reported in cancer. *ARHGEF26-AS1* is downregulated in cancer-associated fibroblasts (CAFs) of HGSOC patients compared with normal ovarian fibroblasts (NOFs) [62]. As CAFs promote cancer metastasis [62], our results showing that ILK depletion leads to *ARHGEF26-AS1* downregulation suggest additional roles for *ARHGEF26-AS1* in cisplatin-resistant ovarian cancer. Similarly, *MIR7-3HG* upregulation in our analysis contrasts a report showing that *MIR7-3HG* downregulates the tumor suppressor AMBRA1 and prevents MYC dephosphorylation in lung cancer [63]. Nevertheless, our results that *LINC-PINT* was upregulated upon ILK-siRNA transfection is consistent with reports that show that this lncRNA inhibits cell invasion [24]. Interestingly, in osteosarcoma and gastric cancer, *LINC-PINT* inhibits cell invasion, migration, and proliferation by downregulating *miR-21* [64,65]. In ovarian cancer, *LINC-PINT* is downregulated relative to normal ovary cells and tissue samples [66], whereas *miR-21* is upregulated [67]. These observations warrant further investigation to elucidate the role of these lncRNAs in cisplatin-resistant ovarian cancer. 

By using Kaplan–Meier analysis of publicly available mRNA expression (gene chip and RNA-Seq data) we further show that several genes differentially altered upon ILK depletion are significantly associated with survival outcomes in ovarian cancer. Based on expression levels of survival-relevant genes, we identified *CHGA*, *BSN*, *SLC5A1*, *MAPK8IP2*, *NMNAT2*, *SEMA3G*, *ARHGAP23*, *SAG*, *SLC4A8*, *MARVELD3*, *PAX5*, *COL13A1*, *ANKRD22*, *VGF*, *ACTL6B*, *NKAIN1*, *CAMKV*, *LTF*, and *ILK* as potential prognostic markers. Of these genes, we were able to validate *CHGA*, *BSN*, *SLC5A1*, *MAPK8IP2*, *NMNAT2*, *SEMA3G*, *SLC4A8*, *MARVELD3*, *PAX5*, *ANKRD22*, *VGF*, *ACTL6B*, *CAMKV*, and *ILK* by qRT-PCR analysis. In accordance with previous reports, CHGA can exert antiangiogenic effects and inhibit tumor growth in vivo [68]. Overexpression of PAX5 induces apoptosis in multiple myeloma [69], whereas knockdown of *PAX5* increases cell proliferation and cisplatin resistance in esophageal squamous cell carcinoma [70]. Interestingly, *PAX5* promoter hypermethylation has been observed in ovarian cancer [71]. Overexpression of *VGF* inhibits colony formation of ovarian cancer cells, however, *VGF* promoter hypermethylation correlates with better patient survival [72]. In contrast to our results, SEMA3G inhibits cell migration and invasion in glioma [73]. *SLC5A1* promotes growth and proliferation of pancreatic cancer [74]. High expression of SGLT1, which is encoded by *SLC5A1*, is associated with tumor development and poor prognosis in ovarian cancer [75]. In addition, *ANKRD22* promotes progression of non-small-cell lung cancer [76]. However, higher *ANKRD22* expression levels in prostate cancer are associated with longer DFS following radical prostatectomy [77]. Therefore, further studies are required to elucidate the role of these genes and their therapeutic potential in cisplatin-resistant ovarian cancer. 

## 4. Materials and Methods 

### 4.1. Cells and Culture Conditions

The human ovarian epithelial cancer cells, A2780CP20, HEYA8, and HEYA8.MDR, were provided by Dr. Anil K. Sood (MD Anderson Cancer Center, Houston, TX, USA) and have been described elsewhere [78]. A2780 was purchased from the European Collection of Cell Cultures (ECACC, Porton Down, Salisbury, UK), and OV90 and OVCAR3 from the American Type Culture Collection (ATCC, Manassas, VA, USA)—both of which provide authenticated cell lines. OV90CIS and OVCAR3CIS cells were generated by exposing OV90 and OVCAR3 to increasing concentrations of cisplatin (CIS; Sigma-Aldrich, St. Louis, MO, USA). Cells were maintained in RPMI1640 (A2780, A2780CP20, HEYA8, and HEYA8.MDR; HyClone, GE Healthcare Life Sciences, Logan, UT, USA), RPMI1640 + insulin (0.01 mg/mL, OVCAR3 and OVCAR3CIS; Sigma) or M199/MCDB-105 (OV90 and OV90CIS; Gibco, Thermo Fisher Scientific, Grand Island, NY/Sigma) medium supplemented with 10% fetal bovine serum (FBS; HyClone) and 0.1% antibiotic/antimycotic solution (HyClone) at 37 °C in 5% CO_2_ and 95% air. All cell lines were screened for mycoplasma using the LookOut^®^ Mycoplasma PCR detection kit (Sigma), and authenticated by Promega (Madison, WI, USA) and ATCC using Short Tandem Repeat (STR) analysis. In vitro assays were performed at 70%–85% cell density.

### 4.2. Western Blot Analysis

Cell pellets were lysed with ice-cold lysis buffer (1% Triton X, 150 mmol/L NaCl, 25 mmol/L Tris HCl, 0.4 mmol/L NaVO_4_, 0.4 mmol/L NaF, and protease inhibitor cocktail from Sigma) and total protein concentration was determined using Bio-Rad DC Protein Assay reagents (Bio-Rad, Hercules, CA). Protein samples were separated by SDS-PAGE and blotted onto nitrocellulose membranes. The membranes were blocked in either 5% non-fat dry milk (Bio-Rad) or 5% BSA (HyClone) and probed with phospho-ILK (Ser 246; Millipore, Burlington, MA, USA), ILK (Cell Signaling, Danvers, MA, USA), phospho-AKT (Ser 473; Cell Signaling), AKT (Cell Signaling), full caspase-3 (Cell Signaling), or cleaved caspase-3 (Cell Signaling) primary antibodies. Membranes were then incubated with mouse or rabbit IgG horseradish peroxidase (HRP)-linked secondary antibodies (Cell Signaling) followed by enhanced chemiluminescence and autoradiography. Bands were imaged with a FluorChem system (Alpha Innotech Corporation, San Leandro, CA, USA) and the signal intensity of each band was quantified using AlphaEaseFC software. All membranes were reprobed with β-actin monoclonal antibody (Sigma) as a normalizing control. Western blot images with molecular weight markers are shown in Figure S15. 

### 4.3. Immunohistochemistry 

Immunohistochemical (IHC) analysis of p-ILK and ILK was conducted on formalin-fixed paraffin-embedded (FFPE) specimens from chemonaïve serous papillary ovarian cancer (*n* = 10; ages 40–85, mean 64.70 ± 13.320) and normal ovary samples (*n* = 10; ages 43–73, mean 51.70 ± 9.673) provided by the Department of Pathology at UPR-MSC. The study was carried out with approval from the UPR-MSC Institutional Review Board (IRB protocol number A9180115; category 4 exemption; informed consent is not required). A pathologist manually delineated tumor tissue on a representative hematoxylin and eosin (H&E)-stained slide from each paraffin block. Briefly, sections were deparaffinized and rehydrated. After antigen retrieval (Vector Laboratories, Burlingame, CA, USA), endogenous peroxidase was blocked with 3% H_2_O_2_, followed by non-specific protein blocking (Dako, Carpinteria, CA, USA). Human sections were incubated with phospho-ILK (Ser 246; Abcam, Cambridge, MA, USA) or ILK (Cell Signaling) primary antibodies overnight at 4 °C. After washing with PBS, anti-rabbit EnVision+ System-HRP (Dako) was used as a secondary antibody. HRP was detected with 3,3′ diaminobenzidine (DAB; Dako), counterstained with Gill’s No.3 hematoxylin (American MasterTech Scientific, Lodi, CA, USA), and cover slipped with Permount (Fisher Scientific, Fair Lawn, NJ, USA). Scoring of p-ILK and ILK expression was based on the number of positive cells (DAB stained) in each group and quantified in 5 random fields at 20×. One slide per specimen and 10 specimens per group were examined.

### 4.4. Small-Interfering RNA (siRNA) and In Vitro siRNA Transfection 

To target human *ILK* (NM_001014794.2), two siRNAs [5′-GTCAAGTTCTCTTTCCAAT-3′ (ILK-siRNA(1)), and 5′-CTCAATAGCCGTAGTGTAA-3′ (ILK-siRNA(2))] targeting *ILK* mRNA, and a non-silencing negative control siRNA (C-siRNA) were used (Sigma). For in vitro siRNA transfections, siRNAs were mixed with HiPerfect transfection reagent (Qiagen, Valencia, CA) at 1:2 ratio (siRNA: transfection reagent) in serum and antibiotic-free Opti-MEM medium (Gibco). Transfected cells were collected 24 h after treatment for assessment of ILK protein levels by Western blot analysis.

### 4.5. In Vitro Inhibitor Treatment

ILK inhibitor (Cpd22) was purchased from Millipore and dissolved in DMSO (Sigma). For in vitro inhibitor treatment, cells were treated with Cpd22 for 24 h and collected for assessment of p-ILK and ILK protein levels by Western blot analysis. 

### 4.6. In Vitro Cell Viability and Cell Growth

For cell viability assays using siRNAs, A2780, A2780CP20, OVCAR3, OVCAR3CIS, and HEYA8 cells (2 × 10^4^ cells/mL, 3 × 10^4^ cells/mL, or 10 × 10^4^ cells/mL) were seeded into 96-well plates. Twenty-four hours later, siRNA transfection was performed as described above. The transfection mix was replaced with CIS after 24 h of siRNA treatment. Forty-eight hours after CIS treatment, the medium was removed and Alamar blue dye (Invitrogen, Thermo Fisher Scientific, Eugene, OR, USA) was added following the manufacturer’s instructions. Optical density (OD) values were obtained using a plate reader (Bio-Rad) and percentages of cell viability were calculated after blank OD subtraction, taking the untreated cell values as 100% cell viability. For cell viability assays using ILK inhibitor (Cpd22), A2780, A2780CP20, OV90, OV90CIS, OVCAR3, OVCAR3CIS, and HEYA8 cells (2 × 10^4^ cells/mL or 3 × 10^4^ cells/mL) were seeded into 96-well plates. Twenty-four hours later, inhibitor treatment was performed as described above. Seventy-two hours after treatment, cell viability was assessed using Alamar blue dye.

For assessment of cell growth, colony formation assays were performed using Crystal violet dye (Sigma). Briefly, A2780, A2780CP20, OVCAR3, OVCAR3CIS, OV90, and OV90CIS cells (3 × 10^4^ cells/mL or 4.5 × 10^4^ cells/mL) were seeded into 6-well plates. Twenty-four hours later, siRNAs were added to the cells. After treatment, 1000 (A2780CP20) or 2500 (A2780, OVCAR3, OVCAR3CIS, OV90, and OV90CIS) cells were seeded into 10 cm Petri dishes. Seven (A2780, A2780CP20, OVCAR3, and OVCAR3CIS) or ten (OV90 and OV90CIS) days later, colonies were fixed and stained with 0.5% Crystal violet solution in methanol. Colonies of at least 50 cells were scored in five random fields using a light microscope (CKX41; Olympus, Center Valley, PA, USA) with a total magnification of 40×. 

### 4.7. In Vitro Cell Invasion

Cell invasion was analyzed using the matrigel transwell method as previously described [38,79]. A2780CP20, OV90CIS, and OVCAR3CIS cells (3.5 × 10^4^ cells/mL) were seeded into 10 cm Petri dishes and transfected with siRNAs. The next day, matrigel (BD Biosciences, San Jose, CA, USA) in serum-free medium was added onto the upper chambers of 24-well transwell plates and incubated at 37 °C for polymerization. Transfected cells were collected and resuspended in serum-free medium and re-seeded onto the matrigel-coated chamber. Medium containing 10% FBS was added to the lower wells. After 48 h at 37 °C, medium was removed and cells that invaded through the matrigel were fixed and stained using the Protocol Hema 3 Stain Set (Fisher Scientific, Kalamazoo, MI, USA). The invading cells were counted at 20× resolution on an Olympus IX71 microscope equipped with a digital camera (Olympus DP26). Percentages of cell invasion were calculated taking the C-siRNA-transfected cell values as 100% cell invasion.

### 4.8. Caspase-3 Activity

Caspase-3 activity was quantified using the Caspase-3/CPP32 Fluorometric Assay Kit (BioVision, Milpitas, CA, USA) following the manufacturer’s instructions. A2780CP20 cells (2.0 × 10^5^ cells/mL) were seeded into 10 cm Petri dishes and transfected with C-siRNA or ILK-siRNA(2) for 24 h. Seventy-two hours after transfection, floating and attached cells were collected, pellets were lysed, and total protein concentration was determined. Equal amounts of protein were mixed with 2× Reaction Buffer and 1 mM DEVD-AFC substrate in a 96-well plate and incubated at 37 °C for 1 h. Fluorescence intensity at 400 nm excitation and 505 nm emission was measured on a Varioskan Flash reader from Thermo Scientific (Waltham, MA, USA). 

### 4.9. RNA-Seq Analysis

For RNA-Seq analysis, total RNA was isolated from C-siRNA or ILK-siRNA(2)-transfected A2780CP20 cells using the mirVana^TM^ miRNA Isolation Kit (Invitrogen) following the manufacturer’s instructions. RNA quality was verified on all samples with High Sensitivity RNA system on an Agilent 2200 TapeStation instrument (Agilent Technologies, Santa Clara, CA, USA). mRNA was enriched and library was prepared using NEBNext^®^ Poly(A) mRNA Magnetic Isolation and NEBNext^®^ Ultra™ RNA Library Prep (New England Biolabs, Ipswich, MA, USA). Once library construction was complete, qPCR was performed with KAPA SYBR^®^ FAST qPCR in a HiSeq 4000 system (Illumina, San Diego, CA, USA) with a 2 × 150 bp configuration. 

### 4.10. KEGG Pathway Enrichment, Gene Ontology, and Network Analysis

Differentially expressed genes with a log2 fold change ≥1.5 and ≤−1.5 were enriched for their involvement in various biological pathways using KEGG (Kyoto Encyclopedia of Genes and Genomes) Pathway Enrichment. Ontological signatures, mainly biological processes, molecular functions, and cellular components were enriched based on STRING (Search Tool for the Retrieval of Interacting Genes/Proteins) integration of interaction data.

Ingenuity Pathway Analysis (IPA; Ingenuity Systems, Qiagen, Redwood City, CA, USA) software was used to determine the functional networks and pathways associated with differentially expressed genes using a *p*-value cutoff ≤0.001 and a log2 fold change >1.5 or <−1.5. Gene networks and canonical pathways enrichment analysis were performed filtering for all tissues, all cell lines and human species. For the identification of differentially expressed lncRNAs, a *p*-value cutoff ≤0.001 and a log2 fold change >1.0 or <−1.0 were used. 

### 4.11. Survival Analysis

Kaplan–Meier survival analysis was performed using publicly available gene chip and RNA-Seq datasets in Kaplan–Meier (KM) plotter (www.kmplot.com) [25]. For each gene symbol, the probe ID was selected, and ovarian cancer patients were split into high and low expression groups by the median values of mRNA expression. For genes with multiple probes, the best probe was selected. All available datasets were used for survival analysis. Data from ovarian cancer patients, ovarian cancer patients treated with platin, serous ovarian cancer patients, and serous ovarian cancer patients treated with platin were evaluated. KM survival plots for overall survival (OS), progression-free survival (PFS), and relapse-free survival (RFS) were generated with their respective hazard ratios (HR), confidence intervals (CI), and *p*-values (log-rank). *p*-values < 0.05 were considered to be statistically significant. 

### 4.12. SYBR-Green Based qRT-PCR Analysis

For validation of RNA-Seq data, qPCR was performed to assess expression levels of specific genes upon ILK targeting. A custom 96-well plate containing pre-designed primers was purchased from Bio-Rad. Total RNA was isolated from C-siRNA or ILK-siRNA(2)-transfected A2780CP20 cells using the GenElute Mammalian Total RNA Mini Kit (Sigma) following the manufacturer’s instructions, and reverse transcribed using the iScript™ cDNA Synthesis Kit (Bio-Rad). SYBR Green-based qPCR was conducted using SsoAdvanced™ Universal SYBR^®^ Green Supermix (Bio-Rad) and StepOne Plus Real-Time PCR system with the suggested PrimePCR cycling protocol (activation at 95 °C for 2 min, and 40 cycles of 95 °C for 5 s and 60 °C for 30 s).

### 4.13. Statistical Analysis

Graphing and statistical analysis were performed using Student’s *t* test or ANOVA in GraphPad Prism (San Diego, CA, USA) software. *p*-values < 0.05 were considered to be statistically significant. Experiments were performed at least in triplicate. 

## 5. Conclusions

Overall, this study provides further evidence that targeting ILK with siRNA is a plausible approach for ovarian cancer treatment and identifies ILK-regulated genes with potential prognostic and therapeutic value. Further studies are required to fully understand the contribution of ILK downstream effectors to cisplatin resistance in ovarian cancer. 

## Figures and Tables

**Table 1:** KEGG pathway enrichment analysis.

KEGG Term Description	Term ID	Proteins	Hits	*p*-Value	FDR
Metabolic Pathways	1100	1161	1004	0	0
Olfactory Transduction	4740	391	378	2.00 × 10 ^−191^	2.87 × 10 ^−189^
Pathways in Cancer	5200	320	305	1.73 × 10 ^−148^	1.66 × 10 ^−146^
PI3K-AKT Signaling Pathway	4151	336	306	1.26 × 10 ^−133^	9.05 × 10 ^−132^
MAPK Signaling Pathway	4010	251	236	5.65 × 10 ^−111^	3.24 × 10 ^−109^
Neuroactive Ligand–Receptor Interaction	4080	269	246	2.28 × 10 ^−108^	1.09 × 10 ^−106^
HTLV-I Infection	5166	250	229	2.90 × 10 ^−101^	1.19 × 10 ^−99^
Cytokine–Cytokine Receptor Interaction	4060	260	231	2.21 × 10 ^−95^	7.92 × 10 ^−94^
Proteoglycans in Cancer	5205	218	204	7.37 × 10 ^−95^	2.35 × 10 ^−93^
Focal Adhesion	4510	202	190	1.79 × 10 ^−89^	5.13 × 10 ^−88^

**Table 2:** Gene ontology analysis.

GO Term Description	Term ID	Proteins	Hits	*p*-Value	FDR
**Biological Processes**					
Tissue Development	GO:0009888	1416	1330	2.69 × 10 ^−134^	3.62 × 10 ^−130^
Positive Regulation of Macromolecule Biosynthetic Process	GO:0010557	1467	1360	5.37 × 10 ^−124^	3.61 × 10 ^−120^
Cell Development	GO:0048468	1470	1357	1.28 × 10 ^−119^	5.72 × 10 ^−116^
Positive Regulation of RNA Metabolic Process	GO:0051254	1351	1259	3.20 × 10 ^−119^	1.08 × 10 ^−115^
Regulation of Multicellular Organismal Development	GO:2000026	1400	1296	1.43 × 10 ^−116^	3.84 × 10 ^−113^
Positive Regulation of RNA Biosynthetic Process	GO:1902680	1317	1226	5.28 × 10 ^−115^	1.18 × 10 ^−111^
Positive Regulation of Transcription, DNA-Templated	GO:0045893	1294	1204	1.97 × 10 ^−112^	3.31 × 10 ^−109^
Positive Regulation of Nucleic Acid-Templated Transcription	GO:1903508	1294	1204	1.97 × 10 ^−112^	3.31 × 10 ^−109^
Positive Regulation of Cell Communication	GO:0010647	1439	1319	9.10 × 10 ^−110^	1.36 × 10 ^−106^
Neurological System Process	GO:0050877	1116	1051	1.72 × 10 ^−107^	2.31 × 10 ^−104^
**Molecular Functions**					
Adenyl Nucleotide Binding	GO:0030554	1456	1339	8.45 × 10 ^−174^	3.16 × 10 ^−170^
Adenyl Ribonucleotide Binding	GO:0032559	1450	1334	1.60 × 10 ^−173^	3.16 × 10 ^−170^
ATP Binding	GO:0005524	1415	1301	2.12 × 10 ^−168^	2.79 × 10 ^−165^
Signal Transducer Activity	GO:0004871	1436	1309	5.75 × 10 ^−161^	5.67 × 10 ^−158^
Transition Metal Ion Binding	GO:0046914	1355	1236	3.37 × 10 ^−152^	2.66 × 10 ^−149^
Receptor Activity	GO:0004872	1312	1193	6.41 × 10 ^−144^	4.22 × 10 ^−141^
RNA Binding	GO:0003723	1470	1306	1.75 × 10 ^−137^	9.87 × 10 ^−135^
Zinc Ion Binding	GO:0008270	1127	1038	1.56 × 10 ^−134^	7.70 × 10 ^−132^
Signaling Receptor Activity	GO:0038023	1146	1043	7.17 × 10 ^−126^	3.14 × 10 ^−123^
Enzyme Binding	GO:0019899	1255	1127	8.41 × 10 ^−126^	3.32 × 10 ^−123^
**Cellular Components**					
Intrinsic Component of Plasma Membrane	GO:0031226	1471	1335	4.72 × 10 ^−68^	7.28 × 10 ^−65^
Integral Component of Plasma Membrane	GO:0005887	1419	1289	2.85 × 10 ^−66^	1.89 × 10 ^−63^
Neuron Part	GO:0097458	1026	960	3.67 × 10 ^−66^	1.89 × 10 ^−63^
Golgi Apparatus	GO:0005794	1303	1183	2.41 × 10 ^−60^	9.28 × 10 ^−58^
Cell Junction	GO:0030054	1031	954	7.29 × 10 ^−59^	2.25 × 10 ^−56^
Membrane Region	GO:0098589	991	920	1.24 × 10 ^−58^	3.19 × 10 ^−56^
Endoplasmic Reticulum	GO:0005783	1466	1296	1.30 × 10 ^−49^	2.86 × 10 ^−47^
Neuron Projection	GO:0043005	803	746	7.36 × 10 ^−48^	1.42 × 10 ^−45^
Plasma Membrane Region	GO:0098590	827	766	1.06 × 10 ^−47^	1.82 × 10 ^−45^
Extracellular Space	GO:0005615	1194	1069	6.36 × 10 ^−47^	9.81 × 10 ^−45^

**Table 3:** Top 10 upregulated and downregulated genes in ILK-siRNA(2) vs. C-siRNA.

Gene Symbol	Gene Name	Biological Role	Log2 FC	*p*-Value	FDR
**Upregulated**					
*GRIA4*	Glutamate ionotropic receptor AMPA type subunit 4	Excitatory synaptic transmission	9.469	0.00005	0.00101
*SCG3*	Secretogranin III	Platelet degranulation	6.034	0.00005	0.00101
*CHRNB2*	Cholinergic receptor nicotinic beta 2 subunit	Synaptic transmission	4.601	0.00005	0.00101
*XKR7*	XK related 7	Blood group precursor	4.483	0.00005	0.00101
*TOMM40L*	Translocase of outer mitochondrial membrane 40 like	Protein import into mitochondria	4.272	0.00005	0.00101
**Downregulated**					
*ILK*	Integrin linked kinase	Integrin-mediated signal transduction, epithelial to mesenchymal transition, metastasis	−1.853	0.00005	0.00101
*SAG*	S-antigen visual arrestin	Visual perception and surface receptor signaling	−2.074	0.00020	0.00334
*SLC4A8*	Solute carrier family 4 member 8	pH regulation, bicarbonate exchange and cell survival	−2.147	0.00025	0.00403
*TEX41*	Testis expressed 41 (non-protein coding)	Non-coding RNA	−2.895	0.00005	0.00101
*NSG1*	Neuronal vesicle trafficking associated 1	Receptor recycling, protein trafficking, synaptic transmission, apoptosis	−3.229	0.00075	0.01030

**Table 4:** Top 10 upregulated and downregulated lncRNAs in ILK-siRNA(2) vs. C-siRNA.

LNC ID	Log2 FC	*p*-Value	FDR
**Upregulated**			
*MIR7-3HG*	2.375	0.00010	0.00230
*LINC01134*	1.907	0.00035	0.00628
*RP11-64K12.4*	1.818	0.00010	0.00230
*RP11-799D4.4*	1.646	0.00100	0.01455
*RP11-618K13.2*	1.536	0.00005	0.00128
*RP11-380L11.4*	1.470	0.00005	0.00128
**Downregulated**			
*RP11-474G23.2*	−1.029	0.00005	0.00128
*RP11-20G6.3*	−1.056	0.00005	0.00128
*RP11-732A19.8*	−1.576	0.00005	0.00128
*ARHGEF26-AS1*	−2.002	0.00005	0.00128

**Table 5:** Canonical pathways.

Ingenuity Canonical Pathways	−Log *p*-Value	Ratio	Molecules
SAPK/JNK Signaling	2.22	0.0291	DUSP8, MAPK8IP2, MAPK8IP1
NAD Biosynthesis III	1.85	0.2500	NMNAT2
Phototransduction Pathway	1.84	0.0392	RGS9, SAG
NAD Salvage Pathway III	1.75	0.2000	NMNAT2
NAD Biosynthesis from 2-amino-3-carboxymuconate Semialdehyde	1.67	0.1670	NMNAT2
VDR/RXR Activation	1.51	0.0260	COL13A1, CALB1
Reelin Signaling in Neurons	1.37	0.0217	MAPK8IP2, MAPK8IP1
Role of IL-17A in Psoriasis	1.34	0.0769	CCL20
NAD Biosynthesis II (from tryptophan)	1.34	0.0769	NMNAT2

**Table 6:** Top 5 molecular and cellular functions.

Molecular and Cellular Functions	Focus Molecules
Cell Death and Survival	LTF, ILK, STK31, CALB1
Cell Morphology	PAX5, SLC4A8, ILK, LSP1
Cellular Assembly and Organization	GFAP, STON2, GOLGA2, AP3B2, ACTL6B, NPM2, ILK, CPLX1, COL13A1, LMOD1, DCC
Cellular Development	APLN, CDK5R2, FOXP1, CCL20, DCC, ILK, MARVELD3
Cellular Function and Maintenance	ILK, SLC4A8, CCL20, CPLX1, EXOC3L1, CHGA, STON2, GFAP, GOLGA2, DCC

**Table 7:** Validation of target genes by qRT-PCR analysis in ILK-siRNA(2) vs. C-siRNA.

Gene Symbol	FC	*p*-Value
Upregulated		
*ACTL6B*	29.31	0.0112
*CHGA*	7.884	<0.0001
*SLC5A1*	6.524	<0.0001
*PAX5*	5.945	0.0010
*VGF*	5.692	0.0008
*ANKRD22*	3.765	0.0056
*BSN*	3.729	0.0171
*COL13A1*	2.765	0.0724
*MAPK8IP2*	2.403	0.0044
*MARVELD3*	2.318	0.0001
*NMNAT2*	2.283	<0.0001
*CAMKV*	2.238	0.0057
Downregulated		
*NKAIN1*	−0.711	0.1225
*ARHGAP23*	−0.9426	0.4016
*ILK*	−2.539	<0.0001
*SLC4A8*	−2.706	0.0381
*SEMA3G*	−2.822	0.0155

## Supplementary Materials

The following are available online at https://www.mdpi.com/2072-6694/12/4/880/s1, Figure S1: Expression of p-AKT and AKT in ovarian cancer cells, Figure S2: SiRNA-mediated ILK targeting in OV90CIS and HEYA8 cells, Figure S3: SiRNA-mediated ILK targeting in A2780, OVCAR3, and OV90 cells, Figure S4: Inhibitor-mediated ILK targeting in ovarian cancer cells, Figure S5: Effect of a small-molecule ILK inhibitor on p-ILK and ILK expression, Figure S6: Kaplan–Meier plots for gene expression-based overall survival analysis of ovarian cancer patients treated with platin, Figure S7: Kaplan–Meier plots for gene expression-based progression-free survival analysis of ovarian cancer patients treated with platin, Figure S8: Kaplan–Meier plots for gene expression-based overall survival analysis of serous ovarian cancer patients, Figure S9: Kaplan–Meier plots for gene expression-based progression-free survival analysis of serous ovarian cancer patients, Figure S10: Kaplan–Meier plots for gene expression-based overall survival analysis of serous ovarian cancer patients treated with platin, Figure S11: Kaplan–Meier plots for gene expression-based progression-free survival analysis of serous ovarian cancer patients treated with platin, Figure S12: Kaplan–Meier plots for lncRNA expression-based progression-free survival analysis of ovarian cancer patients treated with platin, Figure S13: Kaplan–Meier plots for lncRNA expression-based progression-free survival analysis of serous ovarian cancer patients, Figure S14: Kaplan–Meier plots for lncRNA expression-based progression-free survival analysis of serous ovarian cancer patients treated with platin, Figure S15: Western blot images, Table S1: Differentially expressed genes in ILK-siRNA(2) vs. C-siRNA, Table S2: Differentially expressed lncRNAs in ILK-siRNA(2) vs. C-siRNA, Table S3: Top 5 networks, Table S4: Hazard ratios for overall survival and progression-free survival of ovarian cancer patients based on differential gene expression upon ILK depletion, Table S5: Hazard ratios for overall survival and progression-free survival of ovarian cancer patients treated with platin based on differential gene expression upon ILK depletion, Table S6: Hazard ratios for overall survival and progression-free survival of serous ovarian cancer patients based on differential gene expression upon ILK depletion, Table S7: Hazard ratios for overall survival and progression-free survival of serous ovarian cancer patients treated with platin based on differential gene expression upon ILK depletion, Table S8: Hazard ratios for overall survival and progression-free survival of ovarian cancer patients based on differential lncRNA expression upon ILK depletion, Table S9: Hazard ratios for overall survival and progression-free survival of ovarian cancer patients treated with platin based on differential lncRNA expression upon ILK depletion, Table S10: Hazard ratios for overall survival and progression-free survival of serous ovarian cancer patients based on differential lncRNA expression upon ILK depletion, Table S11: Hazard ratios for overall survival and progression-free survival of serous ovarian cancer patients treated with platin based on differential lncRNA expression upon ILK depletion, and Table S12: Hazard ratios for overall survival and relapse-free survival of ovarian cancer patients based on differential gene expression upon ILK depletion.
