# Supplementary material for: Downstream Effectors of ILK in Cisplatin-Resistant Ovarian Cancer
Source: Cancers (Basel). 2020 Apr 4;12(4):880. doi: 10.3390/cancers12040880 (PMC7226328; doi:10.3390/cancers12040880)
Supplement: Supplementary file 1 [file cancers-12-00880-s001.pdf]

Supplementary Materials

Downstream Effectors of ILK in Cisplatin-Resistant Ovarian Cancer

Jeyshka M. Reyes-González, Blanca I. Quiñones-Díaz, Yasmarie Santana, Perla M. Báez-Vega, Daniel Soto, Fatima Valiyeva, María J. Marcos-Martínez, Ricardo J. Fernández-de Thomas and Pablo E. Vivas-Mejía

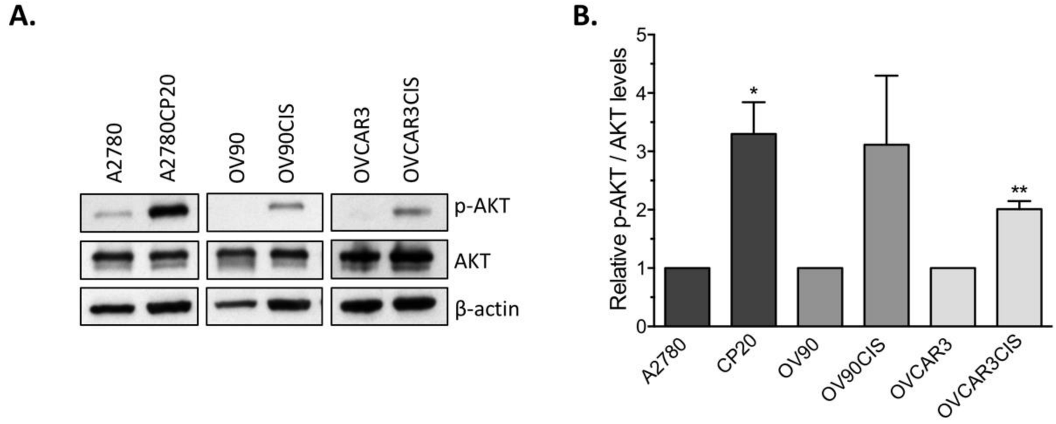

Figure S1. Expression of p-AKT and AKT in ovarian cancer cells. **(A)** Representative Western blots showing the phosphorylated form of AKT (p-AKT) and total AKT protein levels in a panel of ovarian cancer cell lines. **(B)** Densitometric analysis of the band intensities shown in Figure S1A plotted as mean ±SEM (*$p < 0.05$ and ** $p < 0.01$). Phosphorylated AKT / total AKT (p-AKT / AKT) was calculated relative to parental cell lines for each group.

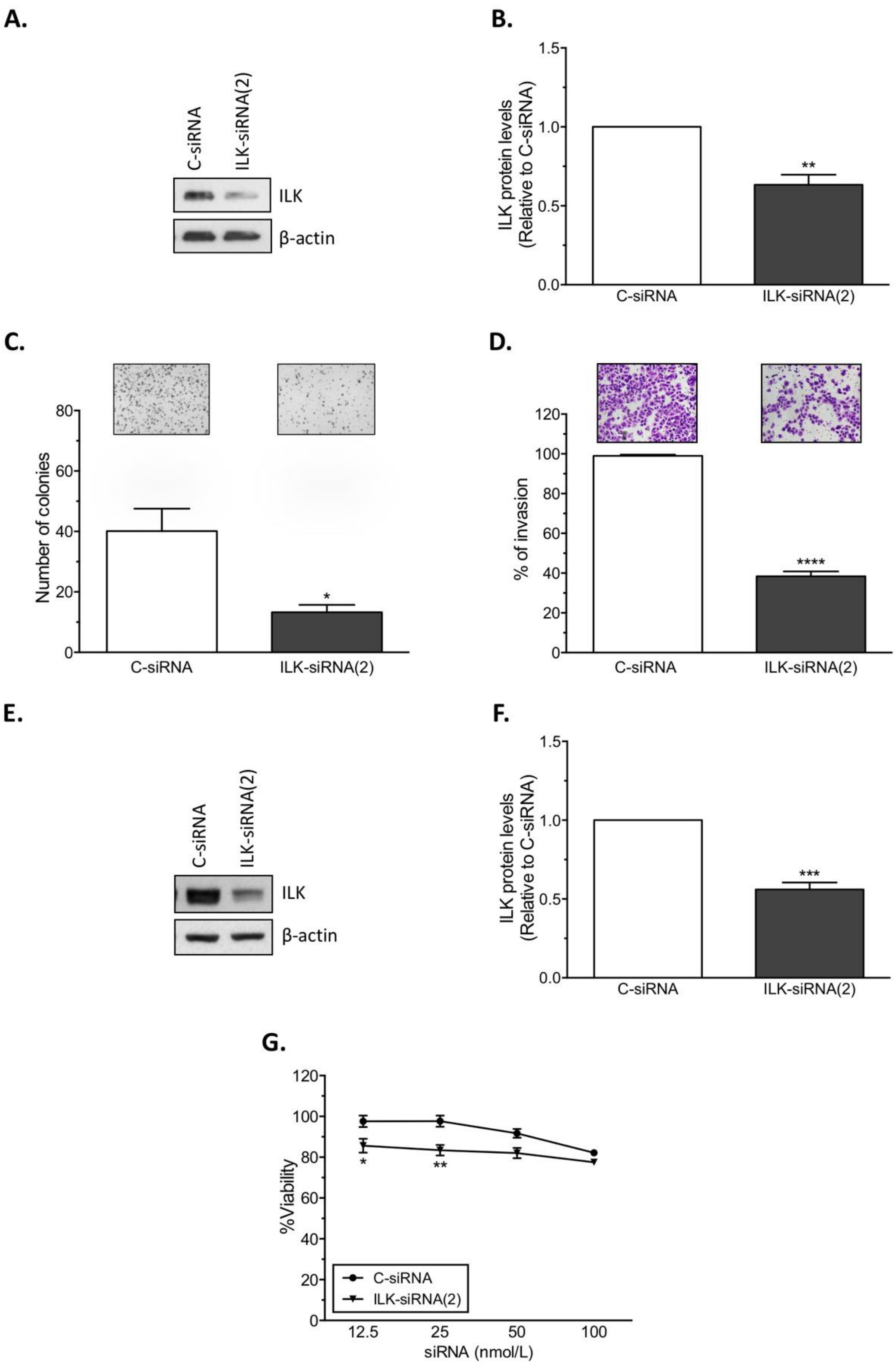

Figure S2. SiRNA-mediated ILK targeting in OV90CIS and HEYA8 cells. A reduction in **(A,B)** ILK protein levels, **(C)** colony formation, and **(D)** invasion ability was observed following ILK-siRNA transfection into OV90CIS cells. A reduction in **(E,F)** ILK protein levels and **(G)** cell viability was observed following ILK-siRNA transfection into HEYA8 cells. Mean ±SEM is shown relative to C-siRNA (* $p < 0.05$, ** $p < 0.01$, *** $p < 0.001$, and **** $p < 0.0001$).

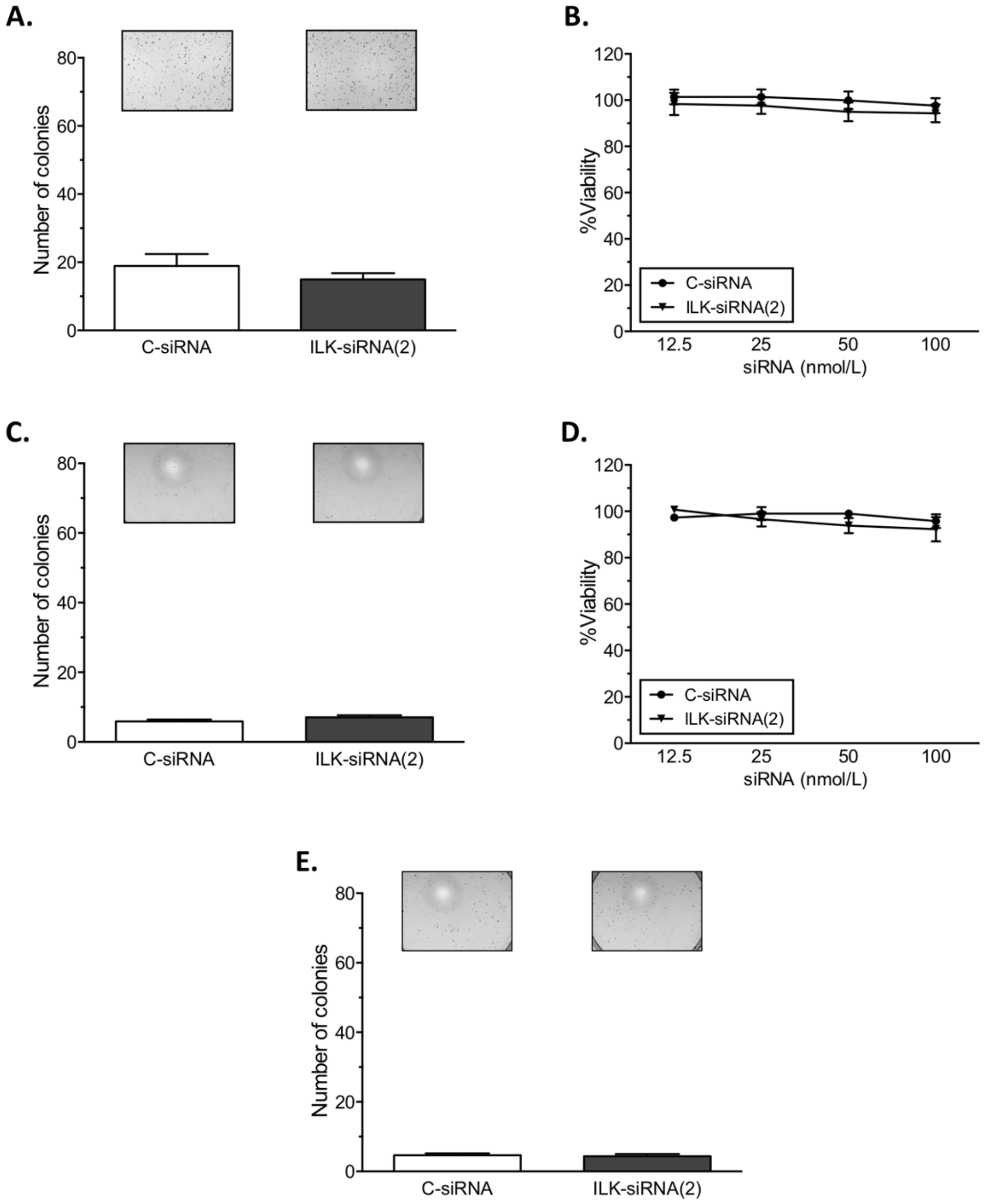

Figure S3. SiRNA-mediated ILK targeting in A2780, OVCAR3, and OV90 cells. SiRNAs were transiently transfected into ovarian cancer cells: **(A,B)** A2780, **(C,D)** OVCAR3, and **(E)** OV90. No significant changes in **(A,C,E)** colony formation or **(B,D)** cell viability were observed following siRNA transfection. Mean ± SEM is shown.

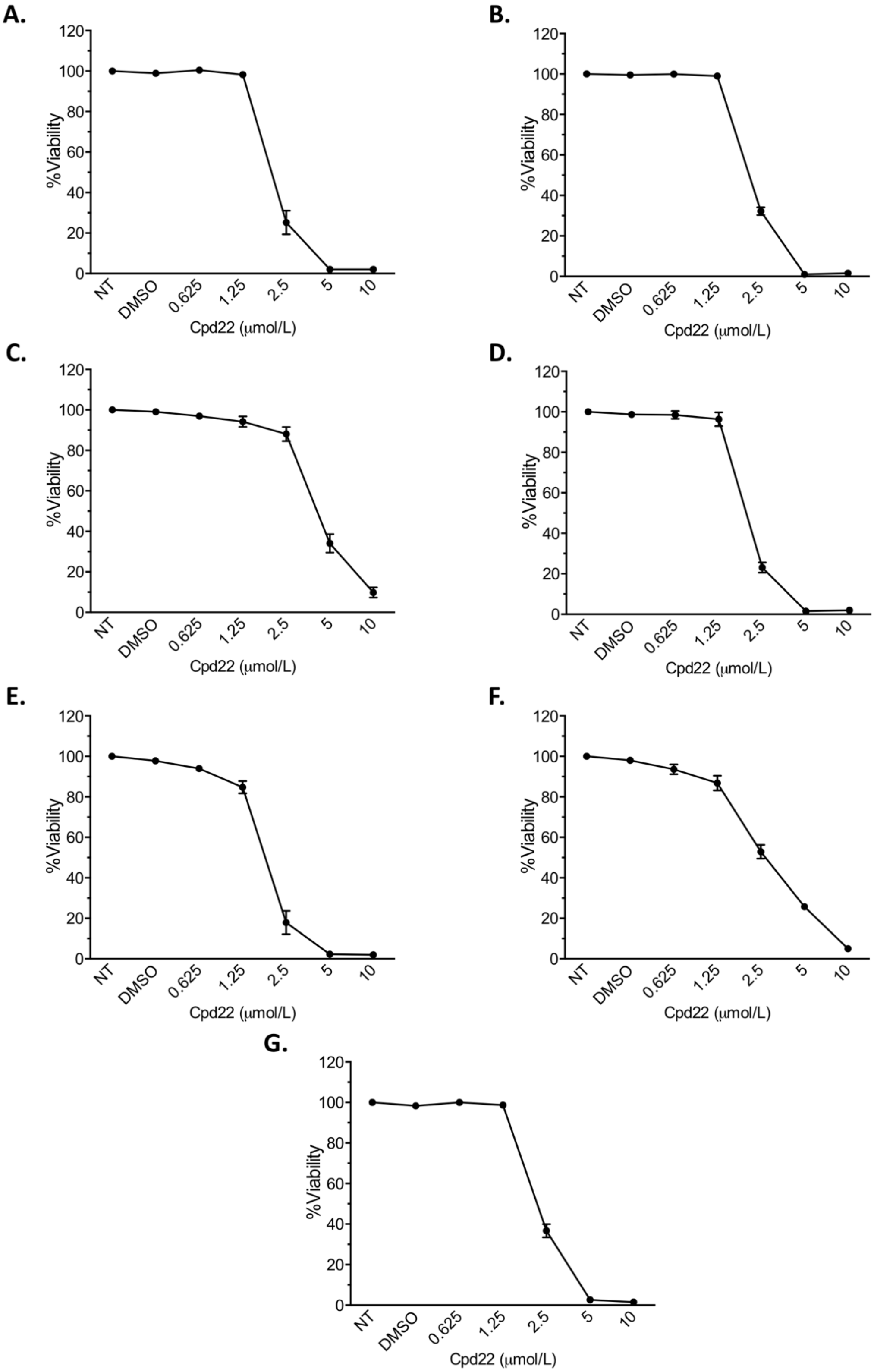

Figure S4. Inhibitor-mediated ILK targeting in ovarian cancer cells. Ovarian cancer cells were treated with ILK inhibitor (Cpd22). A reduction in cell viability was observed for **(A)** A2780CP20, **(B)** OVCAR3CIS, **(C)** OV90CIS, **(D)** A2780, **(E)** OVCAR3, **(F**) OV90, and **(G)** HEYA8 cells. Mean ±SEM is shown.

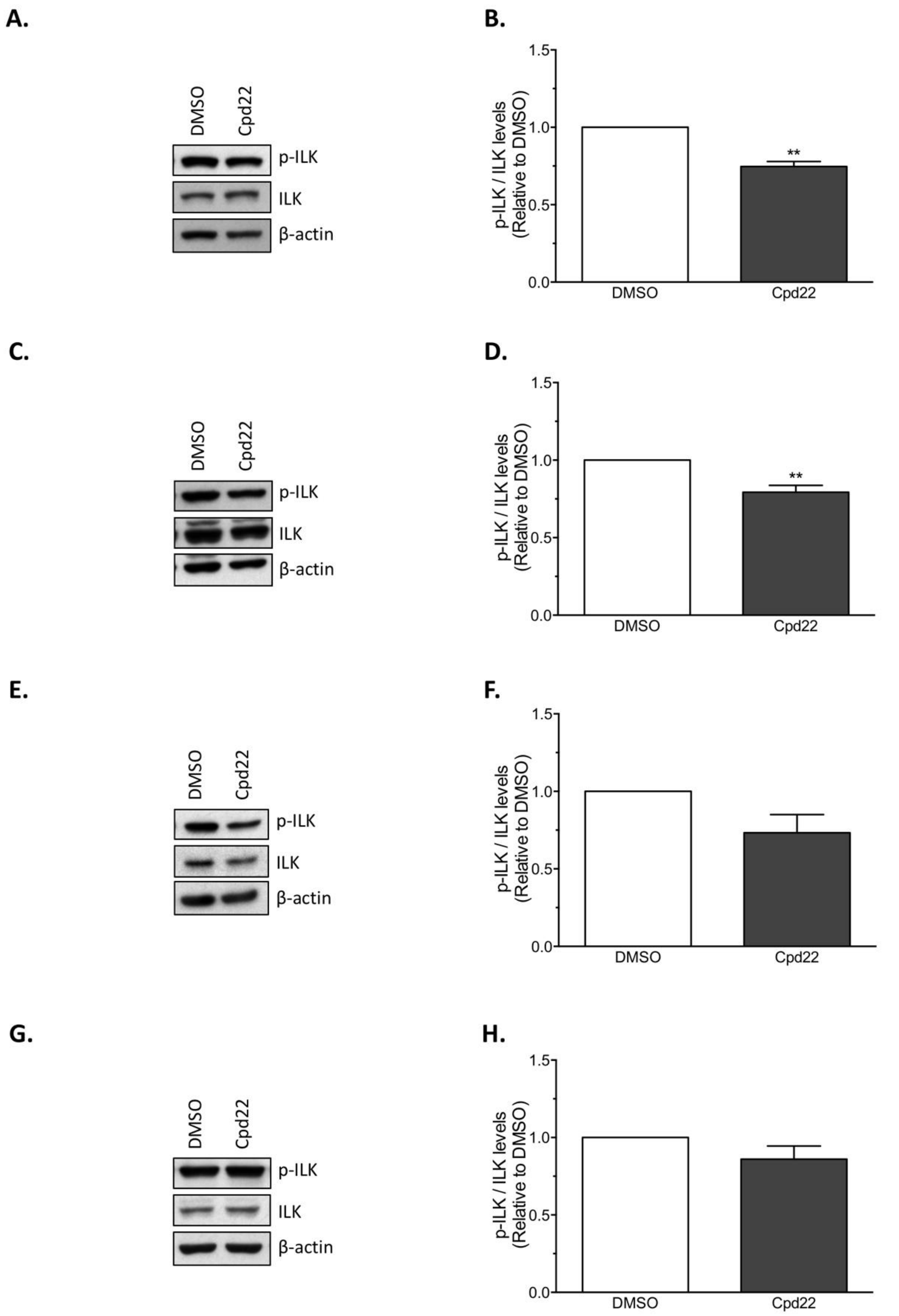

Figure S5. Effect of a small molecule ILK inhibitor on p-ILK and ILK expression. Ovarian cancer cells were treated with ILK inhibitor (Cpd22). Representative Western blots showing the phosphorylated form of ILK (p-ILK) and total ILK protein levels in **(A)** OVCAR3CIS, **(C)** HEYA8, **(E)** A2780CP20, and **(G)** OV90CIS cells. Densitometric analysis of the band intensities shown in **(B)** Figure S5A, **(D)** Figure S5C, **(F)** Figure S5E, and **(H)** Figure S5G plotted as mean ±SEM (** $p < 0.01$). Phosphorylated ILK / total ILK (p-ILK / ILK) was calculated relative to DMSO.

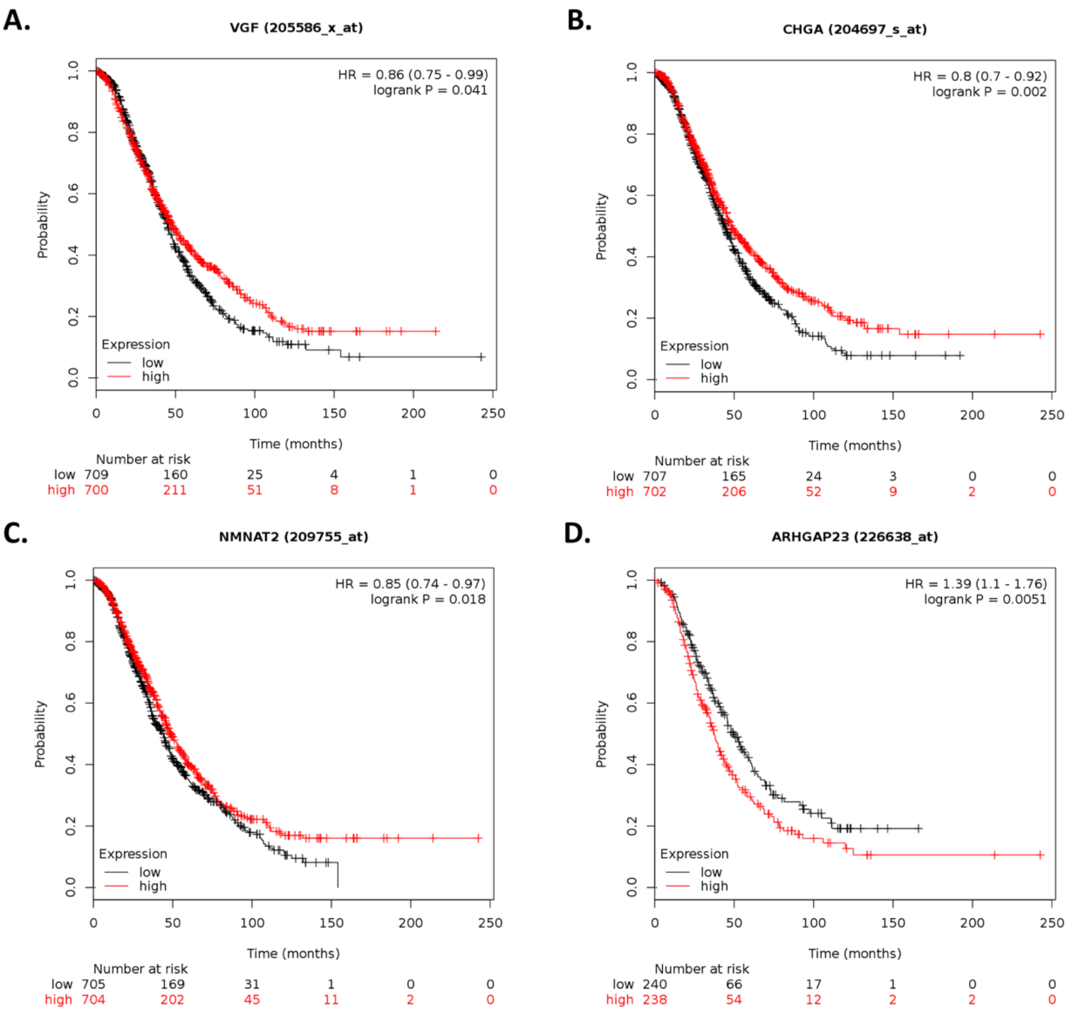

Figure S6. Kaplan-Meier plots for gene expression-based overall survival analysis of ovarian cancer patients treated with platin. Survival plots of ovarian cancer patients treated with platin were generated using Kaplan-Meier plotter (KM plotter). Overall survival (OS) of patients stratified by expression levels of (**A**) VGF, (**B**) CHGA, (**C**) NMNAT2, and (**D**) ARHGAP23 are shown based on gene chip data. *p*-values < 0.05 were considered to be statistically significant.

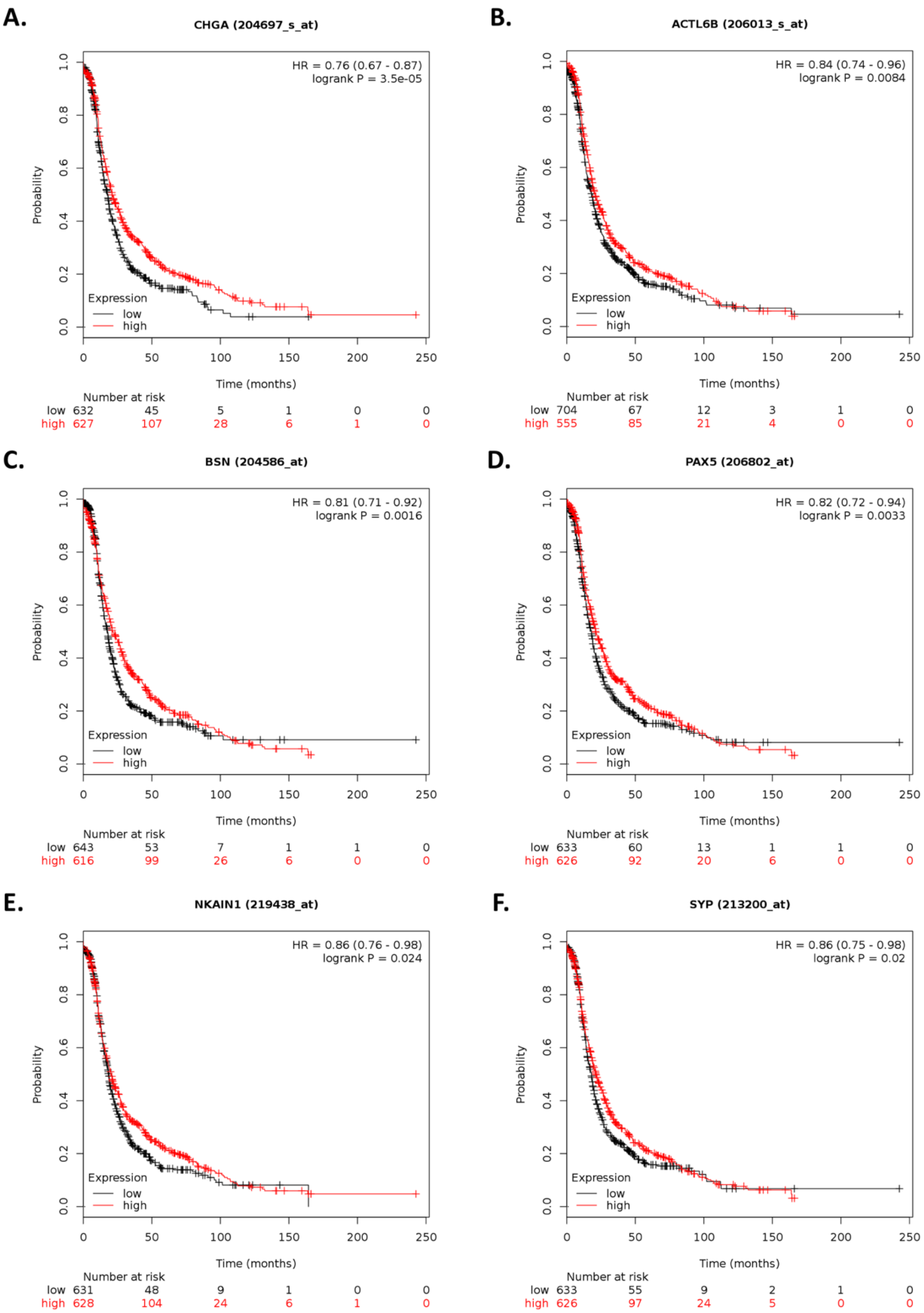

Figure S7. *Cont.*

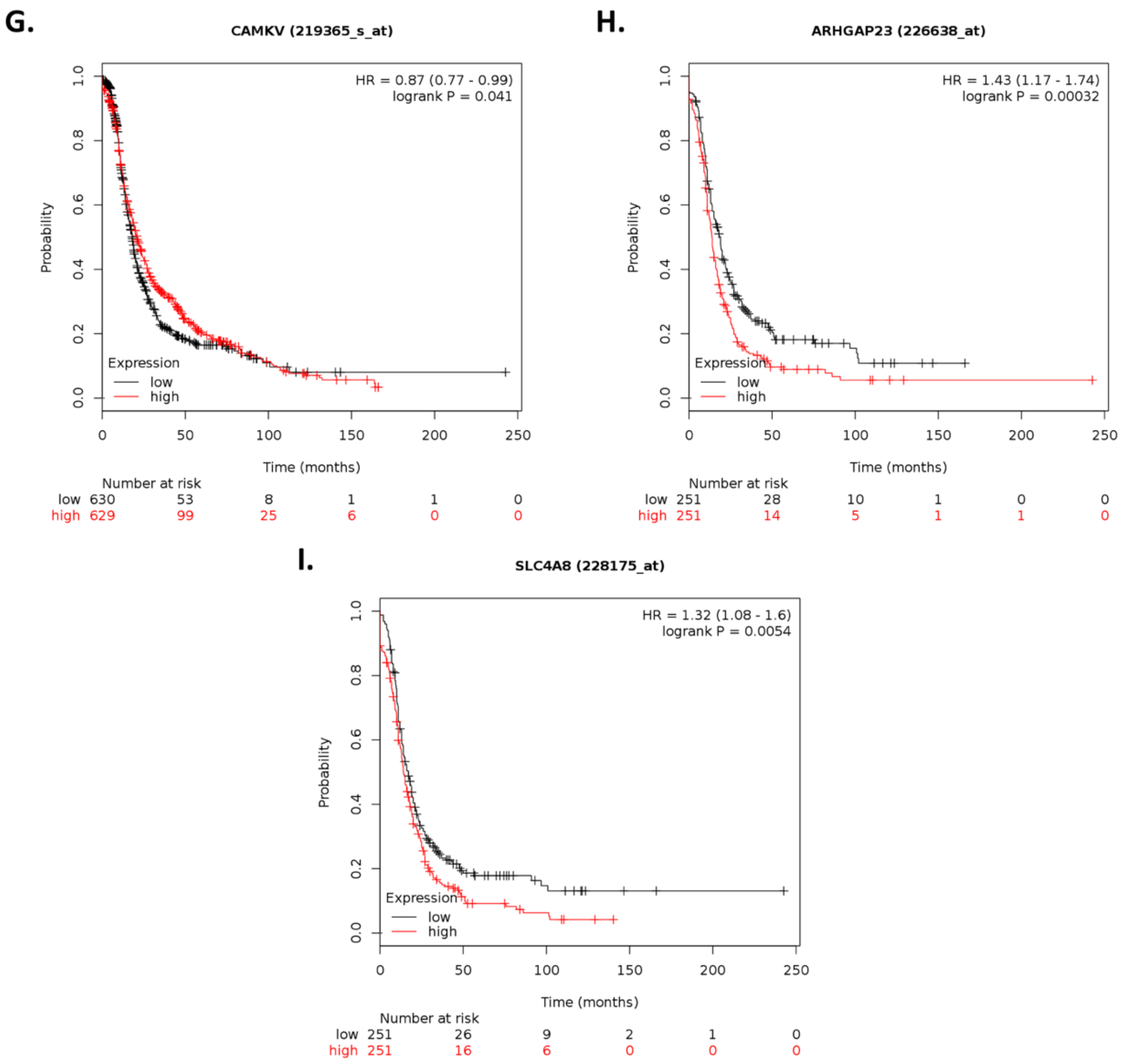

Figure S7. Kaplan-Meier plots for gene expression-based progression-free survival analysis of ovarian cancer patients treated with platin. Survival plots of ovarian cancer patients treated with platin were generated using Kaplan-Meier plotter (KM plotter). Progression-free survival (PFS) of patients stratified by expression levels of (**A**) CHGA, (**B**) ACTL6B, (**C**) BSN, (**D**) PAX5, (**E**) NKAIN1, (**F**) SYP, (**G**) CAMKV, (**H**) ARHGAP23, and (**I**) SLC4A8 are shown based on gene chip data. *p*-values < 0.05 were considered to be statistically significant.

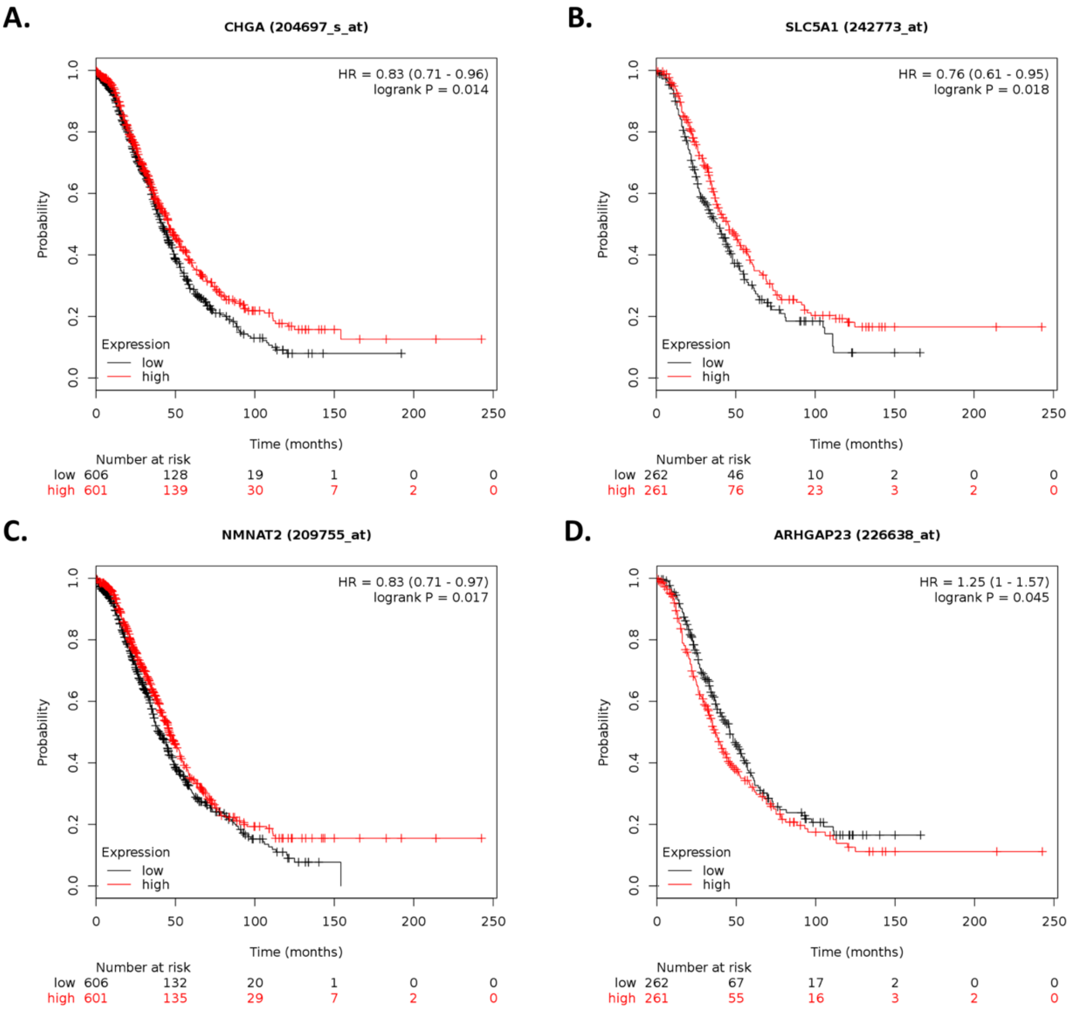

Figure S8. Kaplan-Meier plots for gene expression-based overall survival analysis of serous ovarian cancer patients. Survival plots of serous ovarian cancer patients were generated using Kaplan-Meier plotter (KM plotter). Overall survival (OS) of patients stratified by expression levels of **(A)** CHGA, **(B)** SLC5A1, **(C)** NMNAT2, and **(D)** ARHGAP23 are shown based on gene chip data. p-values < 0.05 were considered to be statistically significant.

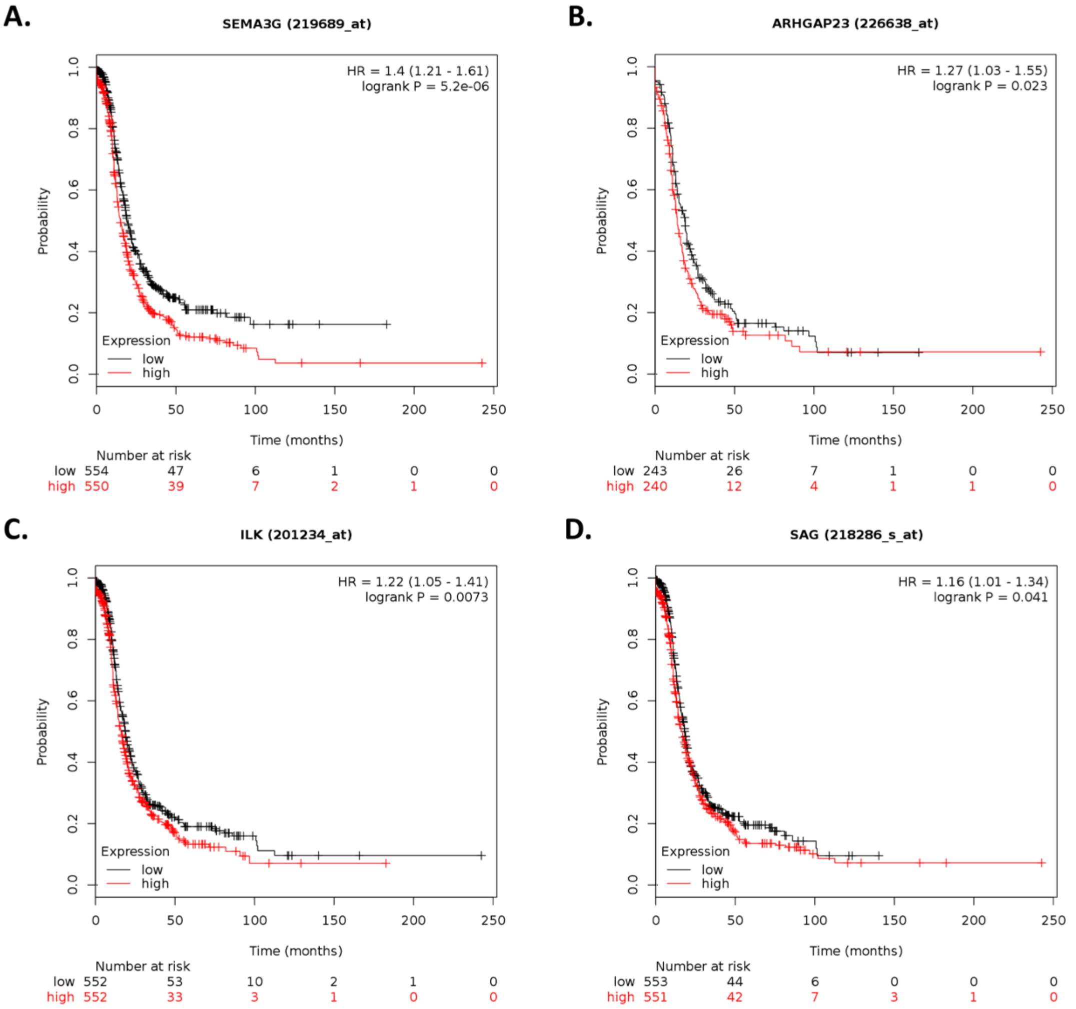

Figure S9. Kaplan-Meier plots for gene expression-based progression-free survival analysis of serous ovarian cancer patients. Survival plots of serous ovarian cancer patients were generated using Kaplan-Meier plotter (KM plotter). Progression-free survival (PFS) of patients stratified by expression levels of **(A)** SEMA3G, **(B)** ARHGAP23, **(C)** ILK, and **(D)** SAG are shown based on gene chip data. P-values <0.05 were considered to be statistically significant.

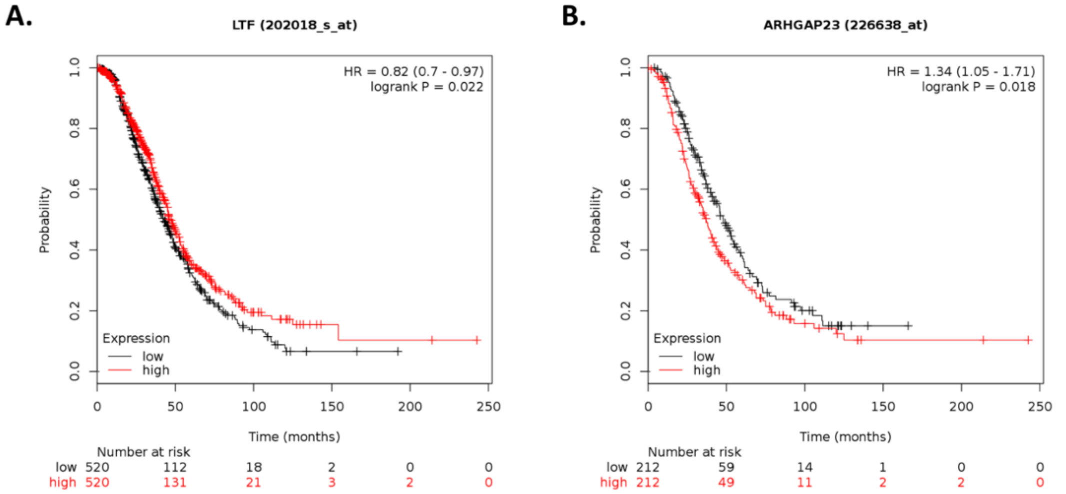

Figure S10. Kaplan-Meier plots for gene expression-based overall survival analysis of serous ovarian cancer patients treated with platin. Survival plots of serous ovarian cancer patients treated with platin were generated using Kaplan-Meier plotter (KM plotter). Overall survival (OS) of patients stratified by expression levels of (**A**) LTF and (**B**) ARHGAP23 are shown based on gene chip data. *p*-values < 0.05 were considered to be statistically significant.

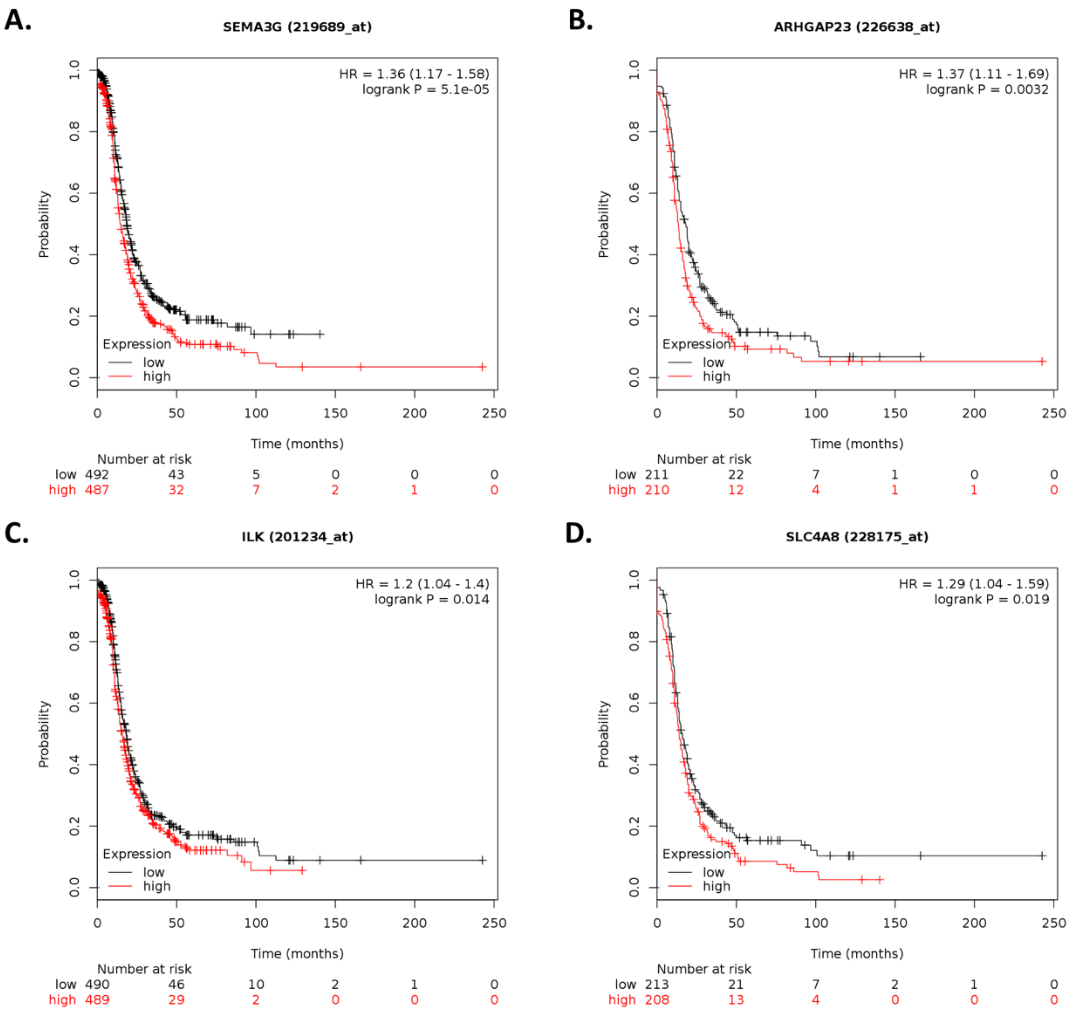

Figure S11. Kaplan-Meier plots for gene expression-based progression-free survival analysis of serous ovarian cancer patients treated with platin. Survival plots of serous ovarian cancer patients treated with platin were generated using Kaplan-Meier plotter (KM plotter). Progression-free survival (PFS) of patients stratified by expression levels of (**A**) SEMA3G, (**B**) ARHGAP23, (**C**) ILK, and (**D**) SLC4A8 are shown based on gene chip data. *p*-values < 0.05 were considered to be statistically significant.

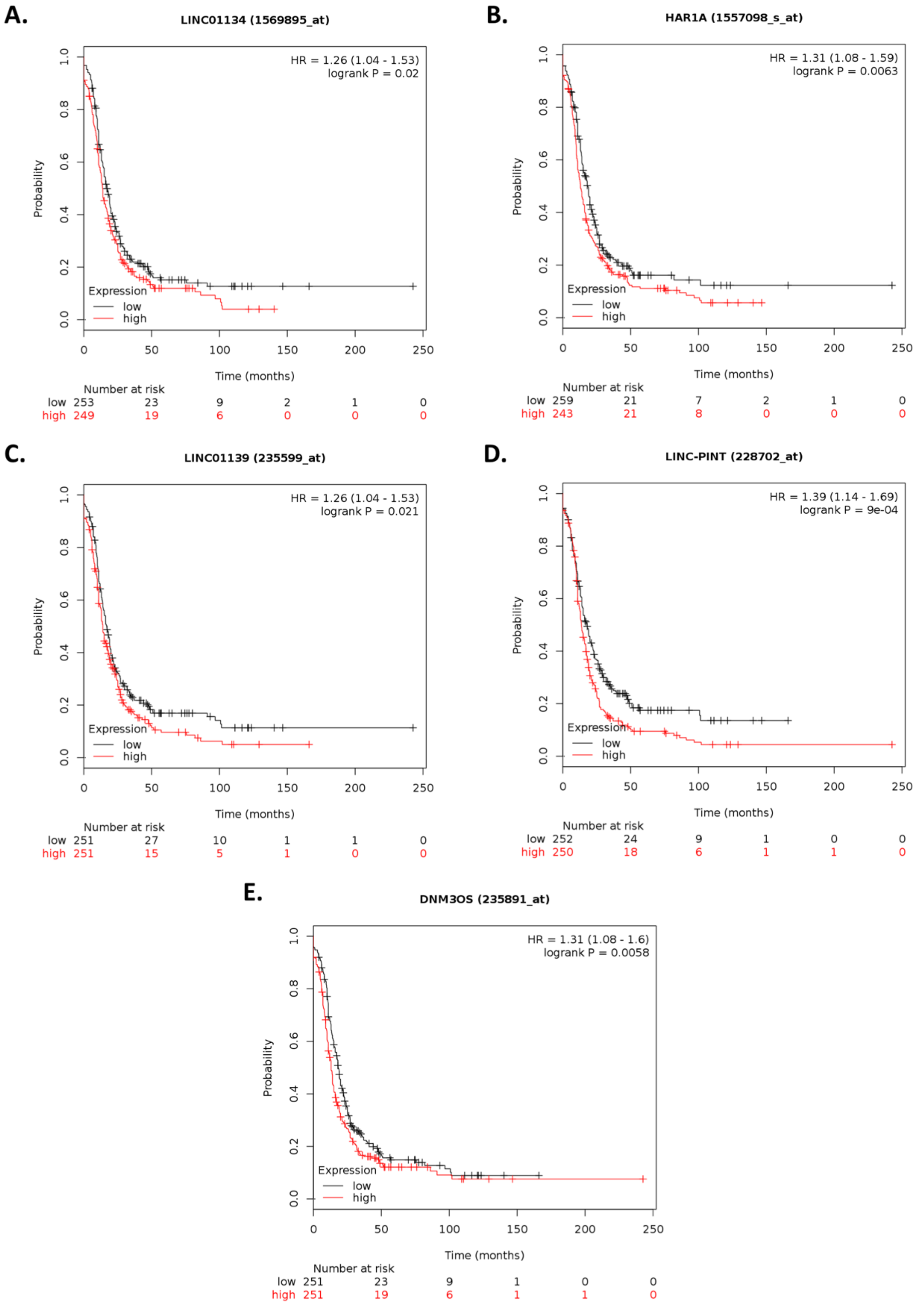

Figure S12. Kaplan-Meier plots for lncRNA expression-based progression-free survival analysis of ovarian cancer patients treated with platin. Survival plots of ovarian cancer patients treated with platin were generated using Kaplan-Meier plotter (KM plotter). Progression-free survival (PFS) of patients stratified by expression levels of (**A**) LINC01134, (**B**) HAR1A, (**C**) LINC01139, (**D**) LINC-PINT, and (**E**) DNM3OS are shown based on gene chip data. *p*-values < 0.05 were considered to be statistically significant.

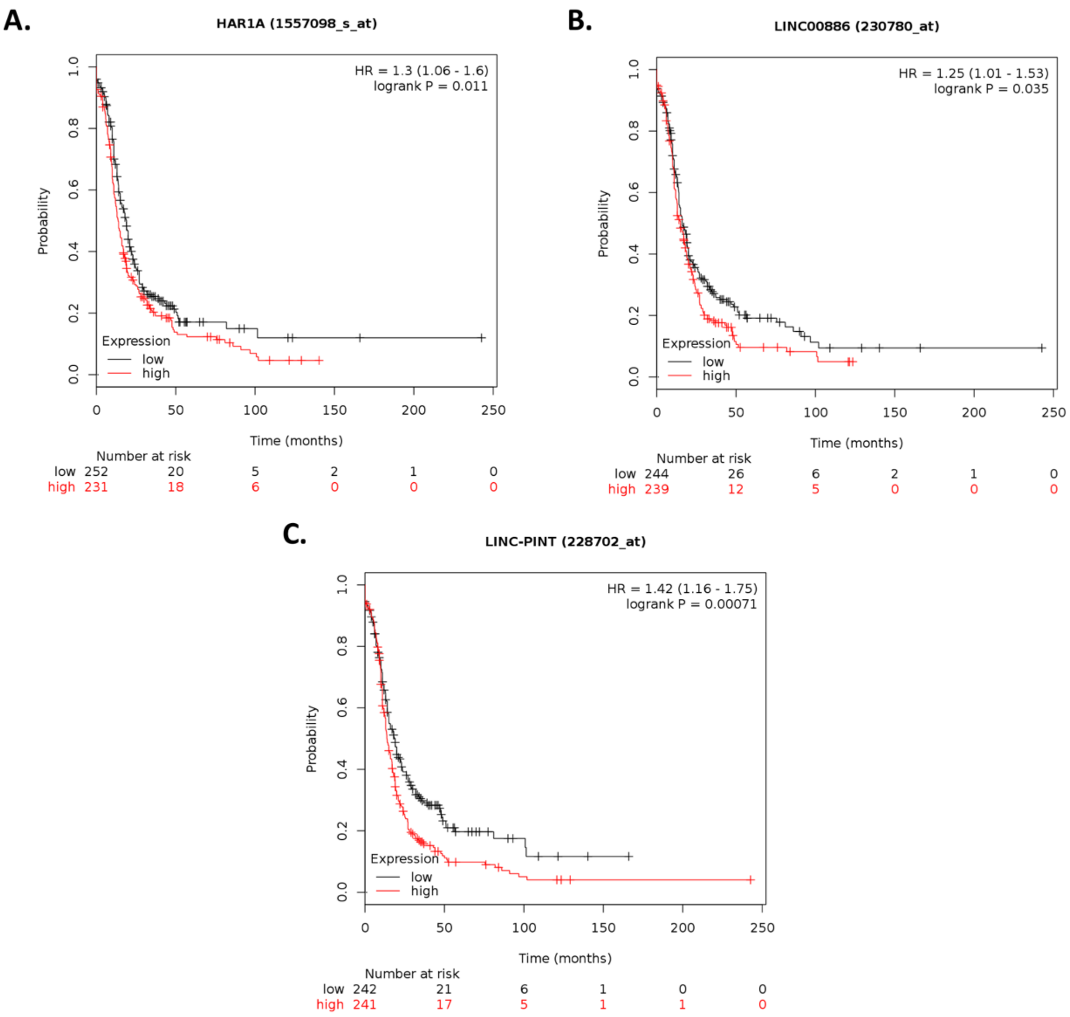

Figure S13. Kaplan-Meier plots for lncRNA expression-based progression-free survival analysis of serous ovarian cancer patients. Survival plots of serous ovarian cancer patients were generated using Kaplan-Meier plotter (KM plotter). Progression-free survival (PFS) of patients stratified by expression levels of (**A**) HAR1A, (**B**) LINC00886, and (**C**) LINC-PINT are shown based on gene chip data. *p*-values < 0.05 were considered to be statistically significant.

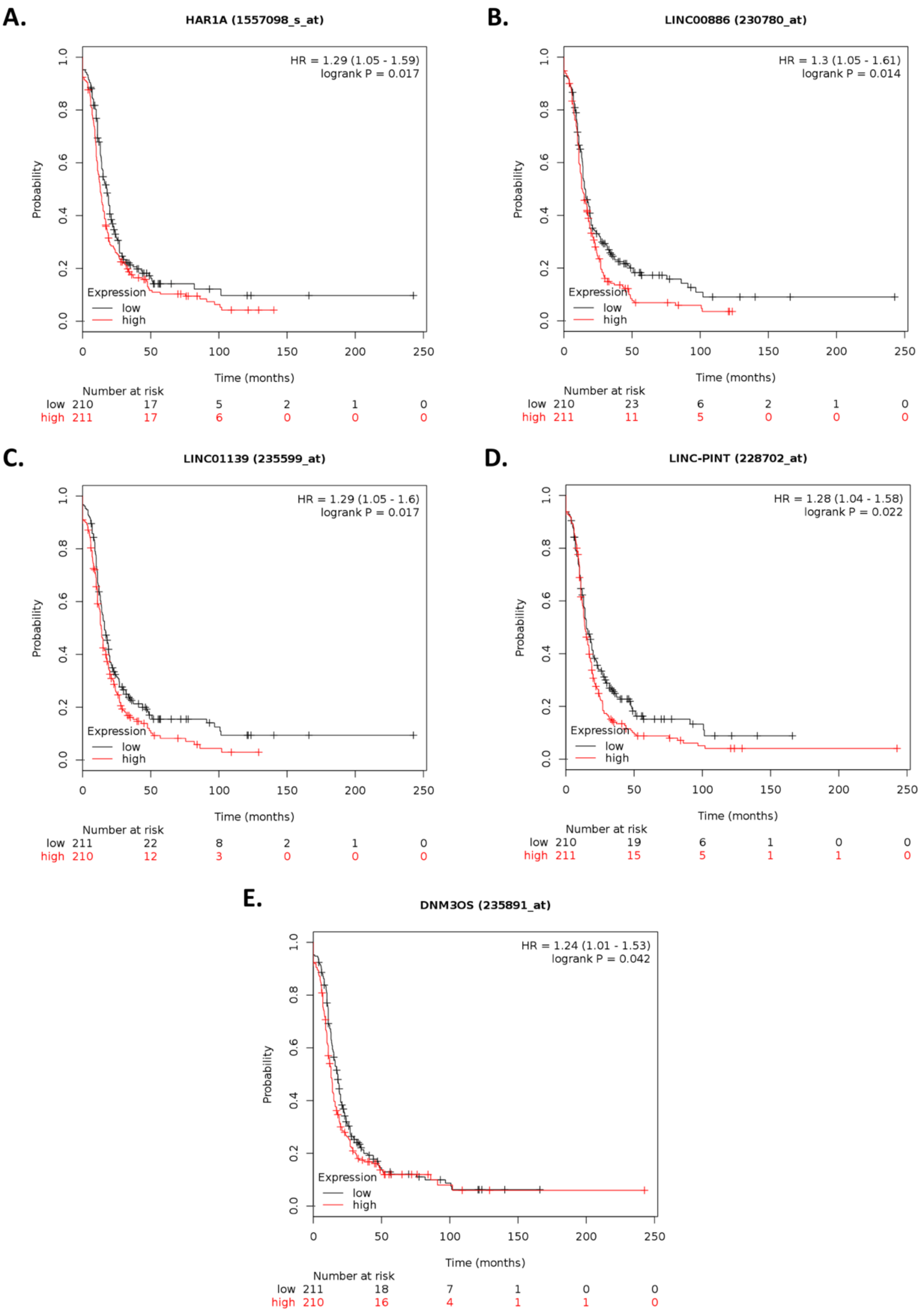

Figure S14. Kaplan-Meier plots for lncRNA expression-based progression-free survival analysis of serous ovarian cancer patients treated with platin. Survival plots of serous ovarian cancer patients treated with platin were generated using Kaplan-Meier plotter (KM plotter). Progression-free survival (PFS) of patients stratified by expression levels of (**A**) HAR1A, (**B**) LINC00886, (**C**) LINC01139, (**D**) LINC-PINT, and (**E**) DNM3OS are shown based on gene chip data. *p*-values < 0.05 were considered to be statistically significant.

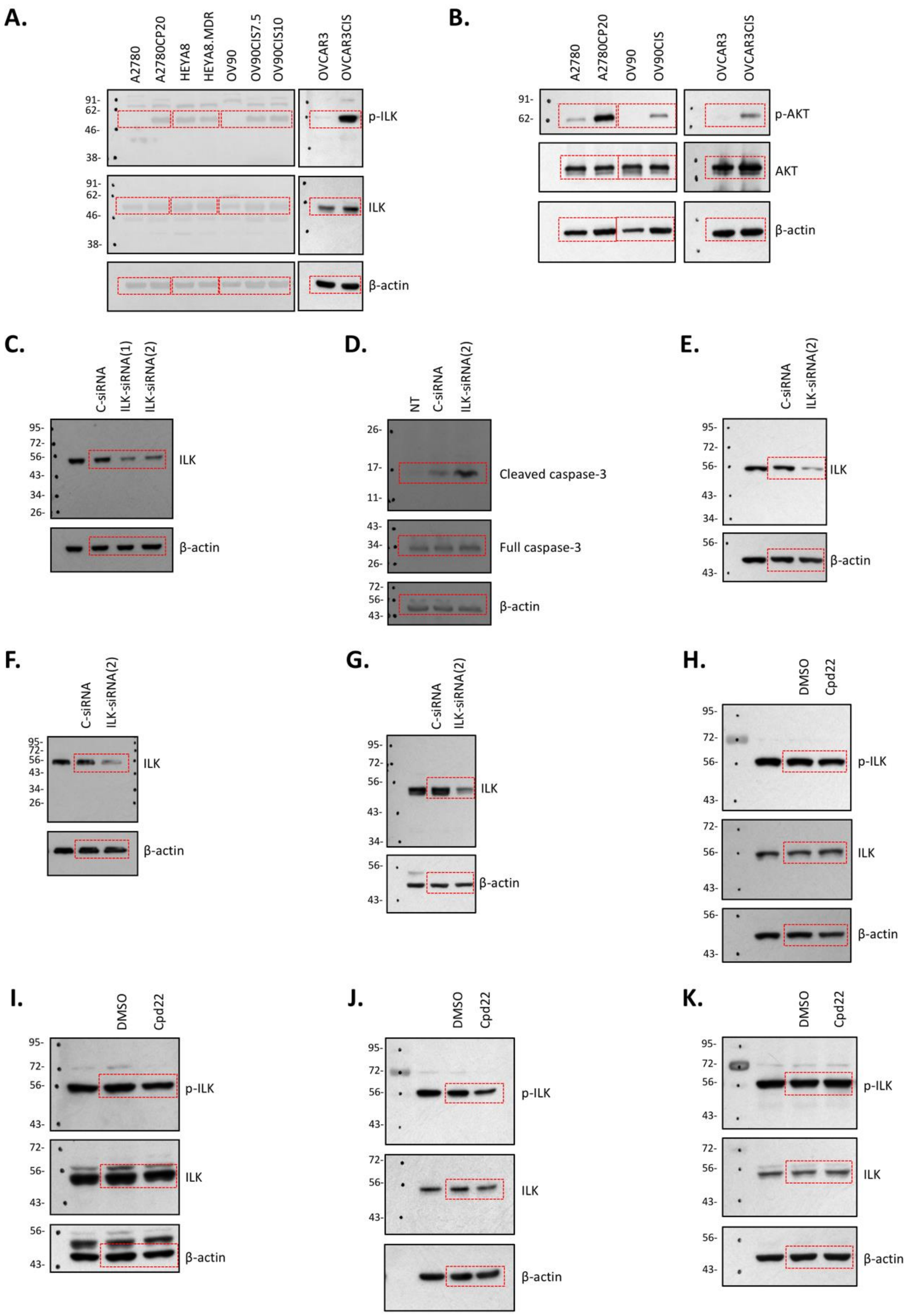

Figure S15. Western blot images. Protein bands and molecular weight markers are shown for **(A)** Figure 1A, **(B)** Figure S1A, **(C)** Figure 2A, **(D)** Figure 2G, **(E)** Figure 3A, **(F)** Figure S2A, **(G)** Figure S2E, **(H)** Figure S5A, **(I)** Figure S5C, **(J)** Figure S5E, and **(K)** Figure S5G.

Table S1. Differentially expressed genes in ILK-siRNA(2) vs. C-siRNA.

Gene Symbol	Gene Name	Log2 FC	P-value	FDR
GRIA4	glutamate ionotropic receptor AMPA type subunit 4	9.469	0.00005	0.00101
SCG3	secretogranin III	6.034	0.00005	0.00101
CHRNB2	cholinergic receptor nicotinic beta 2 subunit	4.601	0.00005	0.00101
XKR7	XK related 7	4.483	0.00005	0.00101
TOMM40L	translocase of outer mitochondrial membrane 40 like	4.272	0.00005	0.00101
FAM172BP	family with sequence similarity 172 member B, pseudogene	4.148	0.00005	0.00101
VGF	VGF nerve growth factor inducible	3.641	0.00005	0.00101
CHGA	chromogranin A	3.482	0.00005	0.00101
RUNDC3A	RUN domain containing 3A	3.476	0.00005	0.00101
CHGB	chromogranin B	3.320	0.00005	0.00101
ACTL6B	actin like 6B	3.295	0.00005	0.00101
BSN	bassoon presynaptic cytomatrix protein	3.261	0.00005	0.00101
TMEM151A	transmembrane protein 151A	3.257	0.00005	0.00101
GOLGA2	golgin A2	3.012	0.00005	0.00101
PAX5	paired box 5	2.945	0.00005	0.00101
CPLX1	complexin 1	2.813	0.00005	0.00101
NKAIN1	sodium/potassium transporting ATPase interacting 1	2.802	0.00005	0.00101
PRSS23	protease, serine 23	2.681	0.00005	0.00101
MIR7-3HG	MIR7-3 host gene	2.602	0.00005	0.00101
SYP	synaptophysin	2.587	0.00005	0.00101
TMEM198	transmembrane protein 198	2.540	0.00005	0.00101
MIGA1	mitoguardin 1	2.493	0.00005	0.00101
SLC5A1	solute carrier family 5 member 1	2.483	0.00020	0.00334
LSP1	lymphocyte-specific protein 1	2.342	0.00010	0.00187
CCL20	C-C motif chemokine ligand 20	2.327	0.00015	0.00262
SLC8A1-AS1	SLC8A1 antisense RNA 1	2.308	0.00005	0.00101
HAPLN2	hyaluronan and proteoglycan link protein 2	2.238	0.00005	0.00101
ZNF385B	zinc finger protein 385B	2.229	0.00005	0.00101
AP3B2	adaptor related protein complex 3 beta 2 subunit	2.178	0.00050	0.00726
COL13A1	collagen type XIII alpha 1 chain	2.151	0.00010	0.00187
MAPK8IP2	mitogen-activated protein kinase 8 interacting protein 2	2.126	0.00005	0.00101
NMNAT2	nicotinamide nucleotide adenylyltransferase 2	2.096	0.00005	0.00101
STK31	serine/threonine kinase 31	2.055	0.00020	0.00334
SEPT7-AS1	SEPT7 antisense RNA 1 (head to head)	2.040	0.00015	0.00262
NPM2	nucleophosmin/nucleoplasmin 2	2.029	0.00015	0.00262
EXOC3L1	exocyst complex component 3 like 1	2.015	0.00005	0.00101
NPTX1	neuronal pentraxin 1	1.998	0.00005	0.00101
APLN	apelin	1.977	0.00005	0.00101
TMEM145	transmembrane protein 145	1.973	0.00005	0.00101
DUSP8	dual specificity phosphatase 8	1.954	0.00005	0.00101
GIPC3	GIPC PDZ domain containing family member 3	1.948	0.00005	0.00101
ANKRD22	ankyrin repeat domain 22	1.946	0.00005	0.00101
CDK5R2	cyclin dependent kinase 5 regulatory subunit 2	1.946	0.00005	0.00101
ELOVL2	ELOVL fatty acid elongase 2	1.944	0.00005	0.00101
NID1	nidogen 1	1.906	0.00005	0.00101
CAMKV	CaM kinase like vesicle associated	1.893	0.00005	0.00101
ARMCX2	armadillo repeat containing, X-linked 2	1.829	0.00005	0.00101
DCC	DCC netrin 1 receptor	1.808	0.00090	0.01190
GPNMB	glycoprotein nmb	1.800	0.00005	0.00101
GFAP	glial fibrillary acidic protein	1.792	0.00090	0.01190
LTF	lactotransferrin	1.783	0.00015	0.00262
PLA2G4C	phospholipase A2 group IVC	1.723	0.00005	0.00101
DISP2	dispatched RND transporter family member 2	1.719	0.00035	0.00538

TMEM121B	transmembrane protein 121B	1.718	0.00005	0.00101
MARVELD3	MARVEL domain containing 3	1.716	0.00065	0.00905
SLC6A17	solute carrier family 6 member 17	1.712	0.00005	0.00101
LMOD1	leiomodin 1	1.710	0.00005	0.00101
CALB1	calbindin 1	1.671	0.00075	0.01030
KDF1	keratinocyte differentiation factor 1	1.644	0.00025	0.00403
STON2	stonin 2	1.630	0.00005	0.00101
LINC01134	long intergenic non-protein coding RNA 1134	1.629	0.00100	0.01290
RGS9	regulator of G protein signaling 9	1.615	0.00080	0.01080
PNRC1	proline rich nuclear receptor coactivator 1	1.605	0.00005	0.00101
MAPK8IP1	mitogen-activated protein kinase 8 interacting protein 1	1.584	0.00005	0.00101
SLFN5	schlafen family member 5	1.567	0.00005	0.00101
FOXP1	forkhead box P1	1.536	0.00025	0.00403
LINC01273	long intergenic non-protein coding RNA 1273	1.523	0.00005	0.00101
PDCD4	programmed cell death 4	1.523	0.00005	0.00101
HIPK3	homeodomain interacting protein kinase 3	-1.507	0.00005	0.00101
SEMA4A	semaphorin 4A	-1.536	0.00010	0.00187
SEMA3G	semaphorin 3G	-1.625	0.00045	0.00664
ARHGAP23	Rho GTPase activating protein 23	-1.853	0.00065	0.00905
ILK	integrin linked kinase	-1.853	0.00005	0.00101
SAG	S-antigen visual arrestin	-2.074	0.00020	0.00334
SLC4A8	solute carrier family 4 member 8	-2.147	0.00025	0.00403
TEX41	testis expressed 41 (non-protein coding)	-2.895	0.00005	0.00101
NSG1	neuronal vesicle trafficking associated 1	-3.229	0.00075	0.01030

Table S2. Differentially expressed lncRNAs in ILK-siRNA(2) vs. C-siRNA.

LNC ID	Log2 FC	P-value	FDR
MIR7-3HG	2.375	0.00010	0.00230
LINC01134	1.907	0.00035	0.00628
RP11-64K12.4	1.818	0.00010	0.00230
RP11-799D4.4	1.646	0.00100	0.01455
RP11-618K13.2	1.536	0.00005	0.00128
RP11-380L11.4	1.470	0.00005	0.00128
RP11-206M11.7	1.455	0.00005	0.00128
RP11-88I18.2	1.404	0.00005	0.00128
RP11-284F21.9	1.401	0.00005	0.00128
HAR1A	1.387	0.00020	0.00404
LINC00886	1.244	0.00010	0.00230
RP11-284F21.7	1.234	0.00005	0.00128
CTD-2587H24.10	1.203	0.00015	0.00320
RP3-332B22.1	1.198	0.00005	0.00128
HLA-F-AS1	1.190	0.00005	0.00128
DLGAP1-AS1	1.187	0.00005	0.00128
RP11-284F21.10	1.177	0.00005	0.00128
LINC01139	1.168	0.00005	0.00128
HOXC-AS1	1.167	0.00005	0.00128
RP11-6N17.4	1.154	0.00005	0.00128
RP11-140H17.1	1.141	0.00005	0.00128
RP11-399O19.9	1.134	0.00005	0.00128
RP11-452L6.5	1.132	0.00005	0.00128
CTC-338M12.1	1.120	0.00005	0.00128
RP11-574F21.2	1.117	0.00005	0.00128
LINC-PINT	1.114	0.00005	0.00128
DNM3OS	1.096	0.00005	0.00128
FRY-AS1	1.068	0.00005	0.00128
AC093627.9	1.052	0.00060	0.00949

RP6-65G23.3	1.041	0.00005	0.00128
RP11-430L3.1	1.026	0.00020	0.00404
RP11-1C8.4	1.019	0.00015	0.00320
NEAT1	1.018	0.00005	0.00128
RP11-474G23.2	-1.029	0.00005	0.00128
RP11-20G6.3	-1.056	0.00005	0.00128
RP11-732A19.8	-1.576	0.00005	0.00128
ARHGEF26-AS1	-2.002	0.00005	0.00128

Table S3. Top 5 networks.

Top Diseases and Functions	Score	Molecules in Network†
Nervous System Development and Function, Organ Morphology, Organismal Development	23	AR, **ARMCX2**, CASP3, CHRNA3, **CHRNB2**, **COL13A1**, COL18A1, CXCL12, DAXX, ESR2, estrogen receptor, FSH, **GFAP**, **HIPK3**, Interferon alpha, ITGA2, ITGA6, LAIR1, **LMOD1**, **MAPK8IP2**, NCOA1, NCOA2, NRXN1, PPARD, PRKAR1A, **SEMA4A**, **SLC5A1**, **SLFN5**, TFAP2A, TGFA, **TMEM121B**, TWIST1, VEGFA, **VGF**, **ZNF385B**
Cell Death and Survival, Cancer, Immunological Disease	23	**ANKRD22**, BIRC5, **CAMKV**, CCND1, **CHGA**, **CHGB**, COL18A1, CREBBP, CSF1R, EIF4A, EP300, **FOXP1**, **GRIA4**, HAMP, KAT5, mir-486, miR-486-5p (and other miRNAs w/seed CCUGUAC), NANOG, **NPTX1**, NR5A2, **PAX5**, PCDH11Y, **PDCD4**, PDX1, PRDM1, PRKCE, **PRSS23**, **SAG**, SFRP4, **SLC4A8**, Smad2/3, SRSF3, STAT5A, **SYP**, WNT1
Cell Death and Survival, Cell-mediated Immune Response, Cellular Movement	16	ADCYAP1, ASAH1, BCR, **CCL20**, CD3 group, CTNNAL1, DEFB103A/DEFB103B, DEFB4A/DEFB4B, ELF3, **ELOVL2**, ERK1/2, **GOLGA2**, IL1RAP, IL1RL2, IL36A, IL36B, IL36G, **ILK**, ITLN1, ITSN1, Jnk, LIMS1, **LSP1**, **LTF**, **MAPK8IP1**, **MARVELD3**, NFkB (complex), P38 MAPK, PARVA, **PLA2G4C**, SDCBP, **STON2**, TLR5, TMSB4, Tnf receptor
Cell-To-Cell Signaling and Interaction, Hematological System Development and Function, Cell Death and Survival	16	AGTR1, **AP3B2**, **APLN**, **BSN**, **CALB1**, CAT, CD86, CD209, COL18A1, **CPLX1**, **DCC**, DEFB4A/DEFB4B, **DUSP8**, Erm, FCER2, **GPNMB**, IL13, IRF4, JAG2, MAP3K1, MAPK8, mir-29, NCF1, **NID1**, PIK3CB, **PNRC1**, S100A8, SBDS, SPP1, STAT1, TGM2, TNF, Tnf receptor, TNFSF14, USF1
Cancer, Cellular Development, Cellular Growth and Proliferation	2	**SEMA3G**, TCF3

†Genes in bold represent focus molecules within networks.

Table S4. Hazard ratios for overall survival and progression-free survival of ovarian cancer patients based on differential gene expression upon ILK depletion.

Gene Symbol†	Probe ID	OS			PFS		
		HR	95% CI	Log-rank P-value	HR	95% CI	Log-rank P-value
TOMM40L	226059_at	0.95	0.78-1.17	0.65	1.25	1.04-1.51	0.02
**CHGA**	204697_s_at	0.79	0.69-0.9	0.00031	0.76	0.67-0.86	1.7E-05
**BSN**	204586_at	0.97	0.85-1.1	0.61	0.88	0.78-1	0.049
CPLX1	223500_at	0.98	0.8-1.2	0.86	1.25	1.03-1.5	0.021
PRSS23	226279_at	1.37	1.12-1.68	0.0024	1.28	1.06-1.54	0.0099
MIR7-3HG	223913_s_at	0.97	0.79-1.19	0.79	1.22	1.01-1.47	0.035
TMEM198	227890_at	1.35	1.1-1.66	0.0037	1.34	1.11-1.62	0.002
**SLC5A1**	242773_at	0.67	0.55-0.82	1E-04	0.97	0.81-1.18	0.79
SLC8A1-AS1	1558920_at	1.12	0.91-1.37	0.28	1.31	1.09-1.58	0.0048
COL13A1	211343_s_at	0.99	0.87-1.13	0.88	1.18	1.04-1.34	0.01
**MAPK8IP2**	205050_s_at	0.87	0.77-0.99	0.042	1	0.88-1.13	0.99
**NMNAT2**	209755_at	0.83	0.73-0.94	0.0034	1.04	0.92-1.18	0.53

STK31	223883_s_at	1.27	1.03-1.55	0.022	1.28	1.06-1.54	0.011
EXOC3L1	1554937_x_at	1.11	0.91-1.36	0.31	1.29	1.07-1.56	0.0073
TMEM145	1553479_at	1.21	0.99-1.49	0.06	1.27	1.05-1.53	0.012
DUSP8	238594_x_at	1.28	1.04-1.57	0.018	1.26	1.04-1.52	0.016
NID1	202007_at	1.18	1.04-1.34	0.012	1.24	1.09-1.4	0.00094
ARMCX2	203404_at	1.12	0.98-1.27	0.094	1.32	1.16-1.5	1.7E-05
GPNMB	201141_at	1.11	0.98-1.27	0.1	1.24	1.1-1.41	0.00062
GFAP	229259_at	1.11	0.91-1.36	0.31	1.3	1.08-1.57	0.0052
**PLA2G4C**	209785_s_at	0.87	0.76-0.99	0.034	0.96	0.84-1.08	0.48
DISP2	229579_s_at	1.3	1.06-1.6	0.01	1.33	1.1-1.6	0.0031
LMOD1	203766_s_at	1.2	1.06-1.37	0.0045	1.11	0.98-1.26	0.11
STON2	227461_at	1.07	0.88-1.31	0.49	1.45	1.2-1.76	9.2E-05
LINC01134	1569895_at	1.22	1-1.5	0.054	1.21	1.01-1.46	0.044
PNRC1	209034_at	0.95	0.84-1.08	0.45	1.24	1.09-1.4	0.00087
FOXP1	229844_at	1.26	1.03-1.54	0.025	1.4	1.16-1.69	0.00045
PDCD4	212594_at	0.93	0.82-1.06	0.27	1.18	1.04-1.34	0.01
SEMA4A	219259_at	0.87	0.76-0.99	0.029	0.85	0.75-0.97	0.013
**SEMA3G**	219689_at	1.05	0.92-1.19	0.46	1.17	1.03-1.32	0.017
**ARHGAP23**	226638_at	1.22	1-1.5	0.049	1.43	1.18-1.72	2E-04
**SAG**	218286_s_at	1.04	0.91-1.18	0.58	1.16	1.02-1.32	0.02
**SLC4A8**	228175_at	1.19	0.97-1.46	0.093	1.27	1.05-1.53	0.013

†Genes in bold represent concordance between expression levels upon ILK depletion and survival outcomes.

Table S5. Hazard ratios for overall survival and progression-free survival of ovarian cancer patients treated with platin based on differential gene expression upon ILK depletion.

		**OS**			**PFS**		
**Gene Symbol†**	**Probe ID**	**HR**	**95% CI**	**Log-rank P-value**	**HR**	**95% CI**	**Log-rank P-value**
**VGF**	205586_x_at	0.86	0.75-0.99	0.041	0.88	0.77-1	0.056
**CHGA**	204697_s_at	0.8	0.7-0.92	0.002	0.76	0.67-0.87	3.5E-05
**ACTL6B**	206013_s_at	1.13	0.99-1.3	0.078	0.84	0.74-0.96	0.0084
**BSN**	204586_at	0.95	0.82-1.09	0.43	0.81	0.71-0.92	0.0016
**PAX5**	206802_at	0.97	0.84-1.11	0.64	0.82	0.72-0.94	0.0033
CPLX1	223500_at	1.13	0.9-1.43	0.3	1.23	1.01-1.5	0.036
**NKAIN1**	219438_at	0.93	0.81-1.07	0.29	0.86	0.76-0.98	0.024
PRSS23	226279_at	1.27	1.01-1.6	0.045	1.22	1-1.48	0.045
**SYP**	213200_at	1.04	0.91-1.2	0.57	0.86	0.75-0.98	0.02
TMEM198	227890_at	1.37	1.09-1.73	0.0078	1.19	0.98-1.45	0.072
SLC8A1-AS1	1558920_at	1.14	0.9-1.43	0.28	1.28	1.05-1.55	0.014
**NMNAT2**	209755_at	0.85	0.74-0.97	0.018	1.02	0.9-1.17	0.72
STK31	223883_s_at	1.16	0.92-1.47	0.2	1.23	1.02-1.5	0.034
EXOC3L1	1554937_x_at	1.09	0.87-1.38	0.46	1.3	1.07-1.58	0.0079
DUSP8	238594_x_at	1.25	0.99-1.58	0.06	1.27	1.04-1.54	0.017
NID1	202007_at	1.25	1.09-1.44	0.0015	1.33	1.17-1.52	1.2E-05
**CAMKV**	219365_s_at	0.9	0.78-1.03	0.14	0.87	0.77-0.99	0.041
ARMCX2	203404_at	1.14	0.99-1.31	0.073	1.34	1.18-1.53	1E-05
GPNMB	201141_at	1.03	0.89-1.18	0.72	1.18	1.04-1.34	0.011
GFAP	229259_at	1.05	0.83-1.33	0.68	1.22	1-1.48	0.048
DISP2	229579_s_at	1.43	1.13-1.8	0.0026	1.27	1.05-1.55	0.015
LMOD1	203766_s_at	1.27	1.11-1.46	0.00071	1.14	1-1.3	0.049
STON2	227461_at	1.08	0.86-1.36	0.51	1.22	1.01-1.49	0.042
LINC01134	1569895_at	1.2	0.95-1.52	0.12	1.26	1.04-1.53	0.02
PNRC1	209034_at	1.04	0.9-1.2	0.58	1.33	1.17-1.51	1.5E-05
FOXP1	229844_at	1.4	1.11-1.77	0.0045	1.45	1.2-1.77	0.00015

PDCD4	212594_at	1	0.87-1.15	1	1.22	1.08-1.39	0.0022
SEMA4A	219259_at	0.84	0.73-0.97	0.017	0.8	0.7-0.91	0.00085
**ARHGAP23**	226638_at	1.39	1.1-1.76	0.0051	1.43	1.17-1.74	0.00032
**SLC4A8**	228175_at	1.16	0.92-1.46	0.21	1.32	1.08-1.6	0.0054

†Genes in bold represent concordance between expression levels upon ILK depletion and survival outcomes.

Table S6. Hazard ratios for overall survival and progression-free survival of serous ovarian cancer patients based on differential gene expression upon ILK depletion.

		OS			PFS		
**Gene Symbol†**	**Probe ID**	**HR**	**95% CI**	**Log-rank P-value**	**HR**	**95% CI**	**Log-rank P-value**
SCG3	219196_at	1.12	0.96-1.3	0.15	1.22	1.05-1.4	0.0079
**CHGA**	204697_s_at	0.83	0.71-0.96	0.014	0.88	0.77-1.02	0.094
PRSS23	226279_at	1.25	1-1.57	0.046	1.29	1.05-1.58	0.016
SYP	213200_at	1.02	0.88-1.19	0.8	1.18	1.02-1.36	0.024
TMEM198	227890_at	1.42	1.13-1.77	0.0024	1.41	1.15-1.73	0.00097
**SLC5A1**	242773_at	0.76	0.61-0.95	0.018	0.93	0.76-1.14	0.48
SLC8A1-AS1	1558920_at	1.13	0.9-1.41	0.29	1.35	1.1-1.66	0.0037
HAPLN2	220142_at	0.99	0.85-1.15	0.89	1.16	1.01-1.34	0.039
AP3B2	205678_at	1.04	0.9-1.22	0.57	1.25	1.08-1.44	0.0025
**NMNAT2**	209755_at	0.83	0.71-0.97	0.017	1.06	0.92-1.22	0.42
STK31	223883_s_at	1.23	0.99-1.54	0.066	1.24	1.01-1.52	0.038
EXOC3L1	1554937_x_at	1.07	0.86-1.34	0.54	1.37	1.12-1.68	0.0025
TMEM145	1553479_at	1.18	0.94-1.47	0.15	1.31	1.06-1.6	0.011
DUSP8	238594_x_at	1.34	1.07-1.68	0.0097	1.32	1.07-1.62	0.008
NID1	202007_at	1.14	0.98-1.33	0.083	1.16	1-1.33	0.049
GFAP	229259_at	1.07	0.85-1.34	0.56	1.23	1.01-1.51	0.044
DISP2	229579_s_at	1.25	1-1.57	0.046	1.19	0.97-1.46	0.093
LMOD1	203766_s_at	1.21	1.04-1.41	0.012	1.15	1-1.33	0.053
CALB1	205626_s_at	1.08	0.93-1.26	0.33	1.19	1.03-1.37	0.018
STON2	227461_at	1.09	0.87-1.36	0.45	1.3	1.06-1.6	0.011
PNRC1	209034_at	0.95	0.81-1.1	0.49	1.16	1-1.34	0.048
FOXP1	229844_at	1.29	1.03-1.61	0.025	1.29	1.05-1.59	0.013
SEMA4A	219259_at	0.81	0.7-0.94	0.0068	0.94	0.82-1.09	0.42
**SEMA3G**	219689_at	1.14	0.97-1.32	0.1	1.4	1.21-1.61	5.2E-06
**ARHGAP23**	226638_at	1.25	1-1.57	0.045	1.27	1.03-1.55	0.023
**ILK**	201234_at	1.14	0.98-1.33	0.093	1.22	1.05-1.41	0.0073
**SAG**	218286_s_at	1.05	0.9-1.22	0.55	1.16	1.01-1.34	0.041

†Genes in bold represent concordance between expression levels upon ILK depletion and survival outcomes.

Table S7. Hazard ratios for overall survival and progression-free survival of serous ovarian cancer patients treated with platin based on differential gene expression upon ILK depletion.

		OS			PFS		
**Gene Symbol†**	**Probe ID**	**HR**	**95% CI**	**Log-rank P-value**	**HR**	**95% CI**	**Log-rank P-value**
SCG3	219196_at	1.2	1.02-1.41	0.032	1.27	1.09-1.47	0.0017
RUNDC3A	213439_x_at	1.07	0.91-1.26	0.42	1.2	1.03-1.39	0.018
CHGB	204260_at	0.99	0.84-1.16	0.88	1.19	1.02-1.38	0.023
PRSS23	226279_at	1.23	0.96-1.56	0.096	1.23	1-1.52	0.049
TMEM198	227890_at	1.49	1.17-1.91	0.0013	1.3	1.06-1.61	0.014
SLC8A1-AS1	1558920_at	1.13	0.89-1.44	0.33	1.33	1.08-1.64	0.008
HAPLN2	220142_at	1	0.85-1.18	0.98	1.17	1.0-1.35	0.041
AP3B2	205678_at	1.09	0.92-1.29	0.31	1.23	1.06-1.43	0.0062
EXOC3L1	1554937_x_at	1.12	0.88-1.42	0.37	1.39	1.13-1.72	0.002

DUSP8	238594_x_at	1.31	1.02-1.67	0.031	1.38	1.12-1.7	0.0026
NID1	202007_at	1.18	1-1.39	0.049	1.25	1.08-1.45	0.0033
**LTF**	202018_s_at	0.82	0.7-0.97	0.022	0.93	0.8-1.07	0.3
PLA2G4C	209785_s_at	0.92	0.78-1.09	0.32	1.17	1.01-1.35	0.04
DISP2	229579_s_at	1.29	1.01-1.64	0.04	1.22	0.99-1.51	0.06
LMOD1	203766_s_at	1.26	1.07-1.48	0.0065	1.19	1.03-1.38	0.022
CALB1	205626_s_at	1.15	0.97-1.35	0.1	1.21	1.05-1.41	0.011
PNRC1	209034_at	1.03	0.87-1.22	0.72	1.18	1.01-1.36	0.033
FOXP1	229844_at	1.45	1.13-1.85	0.0028	1.42	1.15-1.76	0.00094
SEMA4A	219259_at	0.75	0.64-0.89	0.00067	0.9	0.78-1.05	0.18
**SEMA3G**	219689_at	1.13	0.96-1.33	0.14	1.36	1.17-1.58	5.1E-05
**ARHGAP23**	226638_at	1.34	1.05-1.71	0.018	1.37	1.11-1.69	0.0032
**ILK**	201234_at	1.14	0.97-1.35	0.12	1.2	1.04-1.4	0.014
**SLC4A8**	228175_at	1.1	0.86-1.4	0.45	1.29	1.04-1.59	0.019

†Genes in bold represent concordance between expression levels upon ILK depletion and survival outcomes.

Table S8. Hazard ratios for overall survival and progression-free survival of ovarian cancer patients based on differential lncRNA expression upon ILK depletion.

		OS			PFS		
**Gene Symbol**	**Probe ID**	**HR**	**95% CI**	**Log-rank P-value**	**HR**	**95% CI**	**Log-rank P-value**
MIR7-3HG	223913_s_at	0.97	0.79-1.19	0.79	1.22	1.01-1.47	0.035
LINC01134	1569895_at	1.22	1-1.5	0.054	1.21	1.01-1.46	0.044
HAR1A	1557098_s_at	1.3	1.06-1.59	0.012	1.4	1.16-1.69	0.00039
LINC01139	235599_at	1.06	0.87-1.3	0.56	1.31	1.09-1.58	0.0044
LINC-PINT	228702_at	1.06	0.87-1.3	0.56	1.51	1.25-1.83	1.6E-05
DNM3OS	235891_at	1.12	0.91-1.37	0.28	1.33	1.1-1.61	0.0026

Table S9. Hazard ratios for overall survival and progression-free survival of ovarian cancer patients treated with platin based on differential lncRNA expression upon ILK depletion.

		OS			PFS		
**Gene Symbol**	**Probe ID**	**HR**	**95% CI**	**Log-rank P-value**	**HR**	**95% CI**	**Log-rank P-value**
LINC01134	1569895_at	1.2	0.95-1.52	0.12	1.26	1.04-1.53	0.02
HAR1A	1557098_s_at	1.15	0.91-1.45	0.23	1.31	1.08-1.59	0.0063
LINC01139	235599_at	1.08	0.86-1.36	0.51	1.26	1.04-1.53	0.021
LINC-PINT	228702_at	1.05	0.83-1.32	0.7	1.39	1.14-1.69	9E-04
DNM3OS	235891_at	1.16	0.92-1.47	0.2	1.31	1.08-1.6	0.0058

Table S10. Hazard ratios for overall survival and progression-free survival of serous ovarian cancer patients based on differential lncRNA expression upon ILK depletion.

		OS			PFS		
**Gene Symbol**	**Probe ID**	**HR**	**95% CI**	**Log-rank P-value**	**HR**	**95% CI**	**Log-rank P-value**
HAR1A	1557098_s_at	1.15	0.92-1.44	0.22	1.3	1.06-1.6	0.011
LINC00886	230780_at	1.13	0.91-1.42	0.27	1.25	1.01-1.53	0.035
LINC-PINT	228702_at	1.05	0.84-1.31	0.68	1.42	1.16-1.75	0.00071

Table S11. Hazard ratios for overall survival and progression-free survival of serous ovarian cancer patients treated with platin based on differential lncRNA expression upon ILK depletion.

		OS			PFS		
**Gene Symbol**	**Probe ID**	**HR**	**95% CI**	**Log-rank P-value**	**HR**	**95% CI**	**Log-rank P-value**
HAR1A	1557098_s_at	1.09	0.85-1.39	0.5	1.29	1.05-1.59	0.017
LINC00886	230780_at	1.15	0.9-1.46	0.26	1.3	1.05-1.61	0.014
LINC01139	235599_at	1.13	0.89-1.44	0.32	1.29	1.05-1.6	0.017
LINC-PINT	228702_at	1.02	0.8-1.3	0.87	1.28	1.04-1.58	0.022
DNM3OS	235891_at	1.14	0.9-1.45	0.28	1.24	1.01-1.53	0.042

Table S12. Hazard ratios for overall survival and relapse-free survival of ovarian cancer patients based on differential gene expression upon ILK depletion.

	OS			RFS		
**Gene Symbol†**	**HR**	**95% CI**	**Log-rank P-value**	**HR**	**95% CI**	**Log-rank P-value**
TMEM151A	1.24	0.96-1.61	0.098	1.47	1.03-2.09	0.031
**PAX5**	0.85	0.65-1.11	0.23	0.67	0.47-0.96	0.028
**COL13A1**	0.95	0.73-1.22	0.67	0.66	0.47-0.95	0.022
**ANKRD22**	0.84	0.65-1.09	0.2	0.59	0.42-0.85	0.0038
**MARVELD3**	0.76	0.59-0.99	0.041	1.12	0.79-1.6	0.51
CALB1	1.25	0.96-1.62	0.092	1.45	1.02-2.07	0.037
HIPK3	0.97	0.75-1.26	0.83	0.68	0.48-0.97	0.033
SEMA4A	0.81	0.62-1.05	0.1	0.6	0.42-0.86	0.0045
SEMA3G	0.83	0.64-1.07	0.16	0.66	0.46-0.94	0.021

†Genes in bold represent concordance between expression levels upon ILK depletion and survival outcomes.
